# Multiple Enzymes Expressed by the Gut Microbiota Can Transform Typhaneoside and Are Associated with Improving Hyperlipidemia

**DOI:** 10.1002/advs.202411770

**Published:** 2025-01-22

**Authors:** Hui Xu, Ru Feng, Meng‐Liang Ye, Jia‐Chun Hu, Jin‐Yue Lu, Jing‐Yue Wang, Heng‐Tong Zuo, Yi Zhao, Jian‐Ye Song, Jian‐Dong Jiang, Yun‐Zhi Zhou, Yan Wang

**Affiliations:** ^1^ State Key Laboratory of Bioactive Substance and Function of Natural Medicines Institute of Materia Medica Chinese Academy of Medical Sciences/Peking Union Medical College Beijing 100050 China; ^2^ Emergency General Hospital National Research Center for Emergency Medicine Beijing 100028 China

**Keywords:** α‐rhamnosidase, β‐glucosidase, gut microbiota, hyperlipidemia, short‐chain fatty acid, typhaneoside

## Abstract

The mechanism of multiple enzymes mediated drug metabolism in gut microbiota is still unclear. This study explores multiple enzyme interaction process of typhactyloside (TYP) with gut microbiota and its lipid‐lowering pharmacological activity. TYP, with bioavailability of only 2.78%, is an active component of *Typha angustifolia L*. and Pushen capsules which is clinically treated for hyperlipidemia. The metabolic process of TYP is identified, and key enzymes involved in TYP metabolism are validated through gene knockout and overexpression techniques. Through overexpressing α‐rhamnosidase (Rha) in *Escherichia coli*, TYP is verified to metabolize into isorhamnetin‐3‐*O*‐neohesperidin (M1) and isorhamnetin‐3‐*O*‐glucoside (M2) after removing rhamnose through Rha. Besides, knockout of β‐glucosidase (Glu) confirms that TYP generates M3 through Glu after removing glucose. Combined with molecular docking, M3 is transformed to generate 3,4‐dihydroxyphenylacetic acid (M4), protocatechuic acid (M5), and 3‐hydroxyphenylacetic acid (M6) through flavonoid reductase (Flr) and chalcone isomerase (Chi). In conclusion, multiple enzymes involved in TYP metabolism (Rha/Glu→Flr→Chi) are identified. Through in vivo experiments, combined use of M3 and M5 also shows excellent anti‐hyperlipidemia efficacy. This is the first study on complex metabolism mechanism and pharmacological activity of natural flavonoids mediated by multiple enzymes, which provide insight to investigate analogous natural products.

## Graphical abstract

Typhactyloside (TYP), with bioavailability of only 2.78%, is an active component of *Typha angustifolia L*. and Pushen capsules which is clinically treated for hyperlipidemia. TYP is metabolized into M1‐M6 by multiple enzymes of gut microbiota: α‐rhamnosidase (Rha), β‐Glucosidase (Glu) Flavonoid reductase (Flr) and Chalcone isomerase (Chi). TYP is metabolized into M3 and M5 to exert anti hyperlipidemia effects.

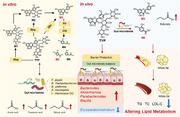

## 1 Introduction

Typhaneoside (TYP) is a flavonoid compound that is an active component of *Typha angustifolia* L. According to the 2020 edition of Chinese Pharmacopoeia, *T. angustifolia* L. has the effects of promoting blood circulation and resolving blood stasis. *T. angustifolia* L. is also clinically used to stop bleeding**^[^** ^1^ ^]^ and treat hyperlipidemia^[^ ^2^ ^]^ in China. In addition, TYP is an active ingredient in a variety of prescriptions, such as Ruan Jian Qing Mai Recipe^[^ ^3^ ^]^ for promoting wound healing, Hugan Qingzhi Formula^[^ ^4^ ^]^ for treating nonalcoholic fatty liver disease, and Shaofu Zhuyu decoction^[^ ^5^, ^6^ ^]^ for treating primary dysmenorrhea. Studies have shown that TYP has numerous pharmacological effects, including antioxidant activity,^[^ ^7^, ^8^ ^]^ anti‐inflammatory effects,^[^ ^9^ ^]^ neuroprotective effects,^[^ ^10^ ^]^ anti‐myocardial ischemia effects,^[^ ^11^ ^]^ therapeutic effects on acute kidney injury,^[^ ^7^ ^]^ hepatoprotective effect on non‐alcoholic fatty liver disease,^[^ ^12^ ^]^ and preventive effects against leukemia.^[^ ^13^ ^]^ Although TYP has a wide range of pharmacological activities, the mechanism of TYP has not been determined, which has hindered further application.

The gut contains a large number of microbes, including bacteria, fungi, and other microorganisms; this community is collectively known as the gut microbiota.^[^ ^14^ ^]^ The gut microbiota has been linked to a variety of diseases, including digestive tract diseases,^[^ ^15^ ^]^ cardiovascular diseases,^[^ ^16^ ^]^ and respiratory diseases.^[^ ^17^ ^]^ Because of the important role of the gut microbiota in disease, the gut microbiota is also important in studying the mechanism of action of drugs. The effects of the gut microbiota on the host are mainly driven by two factors: bacteria and metabolites generated by bacteria.^[^ ^18^ ^]^ On the one hand, bacteria can be classified as beneficial bacteria,^[^ ^19^ ^]^ opportunistic pathogen,^[^ ^20^ ^]^ and harmful bacteria.^[^ ^21^ ^]^ Beneficial bacteria such as *Akkermansia muciniphila* ^[^ ^22^ ^]^ and harmful bacteria, including *Shigella*,^[^ ^23^ ^]^ are examples of these different types. On the other hand, bacterial metabolites mainly include short‐chain fatty acids^[^ ^24^ ^]^ (SCFAs), bile acids,^[^ ^25^ ^]^ polyamine,^[^ ^26^ ^]^ and TMAO.^[^ ^27^ ^]^ Among them, SCFAs are the most essential microbial metabolites and include acetic acid, propionic acid, butyric acid, valerate acid, and caproic acid.^[^ ^28^ ^]^ SCFAs have been implicated in the pathogenesis of many diseases.^[^ ^24^, ^29^, ^30^ ^]^ Therefore, SCFAs are also important targets in the study of drug mechanisms of action.

Natural products generally have a wide range of pharmacological activities when given via oral administration.^[^ ^14^ ^]^ However, the level of exposure of natural products in the blood is very low, and they often have extremely low bioavailability.^[^ ^31^ ^]^ Due to the inevitable contact between natural products and the gut microbiota, the gut microbiota has become a vital direction for studying the mechanism of action of natural products. On the one hand, natural products can be metabolized by the gut microbiota owing to the abundance of diverse types of structures.^[^ ^32^ ^]^ Natural products, on the other hand, also affect the composition of the gut microbiota and bacterial metabolites.^[^ ^33^ ^]^ As a typical flavonoid compound, TYP is a natural product and a wide range of pharmacological activities,^[^ ^7^, ^8^, ^9^, ^10^, ^12^ ^]^ but the mechanism of action is unknown. Accordingly, from the perspective of the gut microbiota, revealing the interaction between TYP and the gut microbiota can help elucidate the mechanism of action of TYP and further promote the application of natural products.

To investigate the molecular mechanism of TYP's pharmacological effects, the interaction between gut microbiota and TYP was studied. The metabolic process of TYP was identified, and specific enzymes involved in the metabolic process were identified and verified through gene knockout and overexpression experiments. Furthermore, in vivo experiments and fecal microbiota transplantation experiments were conducted to verify that the anti‐hyperlipidemia efficacy of TYP was associated with gut microbiota.

## 2 Results

### 2.1 TYP Can Be Metabolized by Gut Microbiota

**Figure 1:**
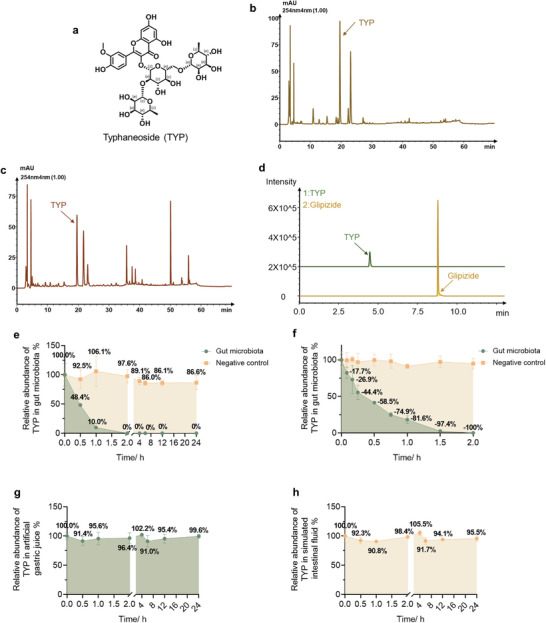
TYP was metabolized rapidly by the gut microbiota. a) Structure of TYP. d) Chromatograms of TYP and glipizide. b) Fingerprint spectra of Cattail Pollen reference crude drug. c) Fingerprint spectra of Pushen capsules. e) Relative abundance of TYP in the gut microbiota within 24 h. f) Relative abundance of TYP in the gut microbiota within 2 h. g) Relative abundance of TYP in artificial gastric juice. h) Relative abundance of TYP in simulated intestinal fluid.

TYP is a flavonoid compound (**Figure** **1**a). TYP is the main active ingredient of Cattail Pollen, and PuShen capsule containing Cattail Pollen is used to treat hyperlipidemia. First, the content of TYP in Cattail Pollen reference crude drug and Pushen capsules was determined by high‐performance liquid chromatography (HPLC). The fingerprint spectra of Cattail Pollen reference crude drug and Pushen capsules are shown in Figure 1b,c, and the standard spectrum of TYP is shown in Figure S1a,b (Supporting Information). The retention time of TYP on HPLC is 19 min. The content of TYP in Cattail Pollen reference crude drug and Pushen capsules is 2.52% and 1.3%, respectively. Then, a method for determining TYP was successfully established via LC‐MS/MS. TYP and glipizide (internal standard, IS) were eluted at 4.5 and 8.8 min, respectively (Figure 1d). According to the pharmacokinetic experiment of TYP, the plasma concentration curve of TYP after oral and intravenous administration is shown in Figure S2a,b (Supporting Information), and the pharmacokinetic parameters are shown in Tables 6 and 7. The oral bioavailability of TYP was about 2.78%. Therefore, the oral efficacy of TYP was closely related to intestinal microbiota. Therefore, the intestinal contents of ob/ob mice were incubated with TYP. Through an in vitro incubation system, we found that TYP was metabolized rapidly by the gut microbiota, but the relative abundance of TYP remained basically unchanged within 24 h in the negative control group (Figure 1e). To obtain more detailed information, the end of the incubation time was set to 2 h. After incubating for 1.5 h, the concentration of TYP decreased by 97.4% (Figure 1f). Therefore, TYP was metabolized rapidly by the gut microbiota. In addition, TYP was not metabolized in artificial gastric juice or simulated intestinal fluid (Figure 1g,h).

### 2.2 Metabolic Characteristics of TYP by Gut Microbiota

Subsequently, a high‐resolution mass spectrometer, LC‐Q‐TOF, was used to identify the metabolites of TYP. With the extension of incubation time, we observed that the abundance of M1–M3 first increased and then decreased, while the abundance of M4–M6 continued to increase (Figures S3c,g,k and S4c,g,k, Supporting Information). Therefore, M1–M3 were speculated to be intermediate metabolites, and M4–M6 were speculated to be the final products. The specific identification processes for M1–M6 were as follows. M1, with *m/z* 623.1616 (Figure S3a,b, Supporting Information), was eluted at 6.2 min (Figure S3c, Supporting Information). Two secondary fragment ions with 314.0429 and 299.0556 were formed from M1, respectively (Figure S3b, Supporting Information). Among them, the secondary fragment ion at *m/z* 314.0429 was formed by removing one rhamnose moiety and one glucose moiety. The sugar chain was removed by homolytic fission, thus forming a radical anion ([M1‐C_6_H_12_O_5_‐C_6_H_9_O_4_·]^−^·, *m/z* = 314.0429). The secondary fragment ion with a *m/z* of 299.0556 was generated by removing one molecule of rhamnose, one molecule of glucose, and a hydroxyl group ([M1‐C_6_H_12_O_5_‐C_6_H_8_O_4_‐O]^−^, *m/z* = 299.0556). The cleavage pathway inferred earlier is shown in Figure S3d (Supporting Information). Combined with the structure of TYP and the mass‐to‐charge ratio of the fragment ions, M1 was speculated to be isorhamnetin‐3‐*O*‐neohesperidin with the molecular formula C_28_H_32_O_16_. Other metabolites showed similar cleavage properties to M1. The quasi‐molecular ion of M2 was [M─H]^−^ with *m/z* 477.1044 (Figure S3e, Supporting Information), and the retention time was 7.4 min (Figure S3g, Supporting Information). The fragment ions formed by M2 were more numerous and included *m/z* 314.0416, *m/z* 271.0622, *m/z* 257.0459, and *m/z* 243.0670 (Figure S3f, Supporting Information). The secondary ion at *m/z* 314.0416 was generated by removing one molecule of glucose, which is a key fragment of isorhamnetin‐3‐*O*‐glucoside.^[^ ^34^ ^]^ The cleavage pathway inferred earlier is shown in Figure S3h (Supporting Information). M2 was speculated to be isorhamnetin‐3‐*O*‐glucoside with a molecular formula C_22_H_22_O_12_. The Ms^n^ spectrogram, EIC, and proposed cleavage pathway of M3 are displayed in Figure S3i–l (Supporting Information). M3 was eluted at 8.6 min (Figure S3k, Supporting Information), and the quasi‐molecular ion of M3 was [M─H]^−^ with *m/z* 315.0512 (Figure S3i, Supporting Information). The fragment ion at *m/z* 300.0271, 271.0624, and 151.0037 was consistent with previous study of isorhamnetin.^[^ ^35^ ^]^ Combining the earlier information, M3 was presumed to be isorhamnetin with the molecular formula C_16_H_12_O_7_.

**Figure 2:**
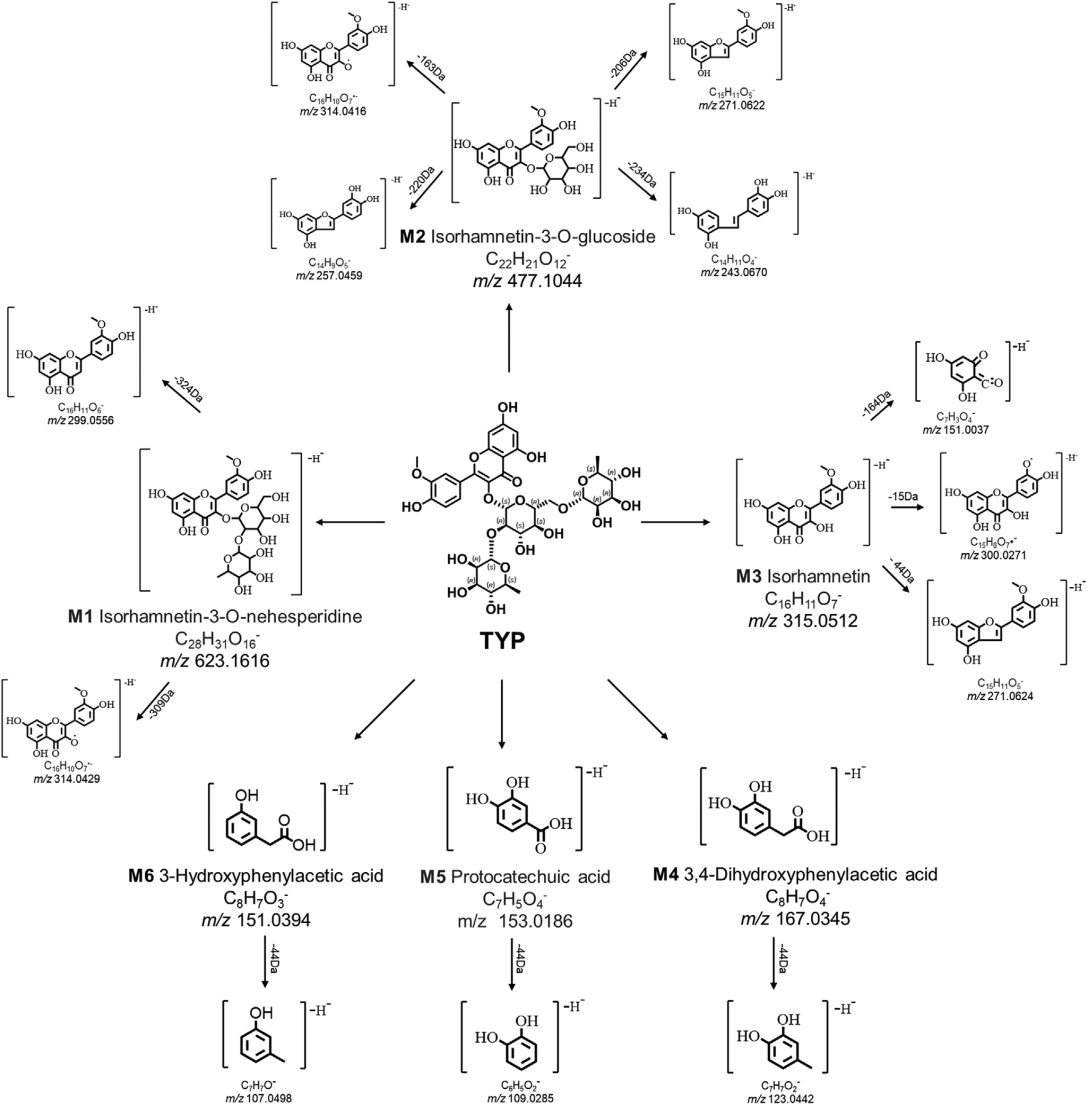
TYP was metabolized was metabolized into six microbiota metabolites.

Due to the smaller size of the molecule, fewer secondary fragment ions were formed from M4 to M6. M4 with *m/z* 167.0345 was eluted at 3.5 min, and the mass spectra of M4 are shown in Figure S4a,b (Supporting Information). The fragment ion at *m/z* 123.0442 was generated by removing one carboxyl group from M4 ([M4‐CO_2_]^−^, *m/z* = 123.0442) (Figure S4b, Supporting Information). The quasi‐molecular ion of M5 was [M─H]^−^ with a *m/z* of 153.0186, and the retention time was 3.4 min (Figure S4e–g, Supporting Information). The secondary ion at *m/z* 109.0285 was in alignment with previous study of protocatechuic acid.^[^ ^36^ ^]^ M6 with *m/z* 151.0394 was eluted at 5.3 min (Figure S4i–k, Supporting Information). The cleavage pathway of M6 was similar to that of M5. The EIC and cleavage pathways inferred earlier are shown in Figure S4 (Supporting Information). Combining the structural formula of TYP and these fragment ions, M4–M6 were inferred to be 3,4‐dihydroxyphenylacetic acid, protocatechuic acid, and 3‐hydroxyphenylacetic acid, respectively. The characteristics of the TYP metabolites identified by LC‐Q‐TOF are summarized (**Figure** **2** and **Table** **1**).

**Table 1:** Characteristics of TYP metabolites in the gut microbiota by LC‐Q‐TOF.

Metabolites	Retention time [min]	Measured *m/z*	Predicted *m/z*	Mass error [ppm]	Predicted molecular formula	Fragment characteristics MS/MS
M1	6.2	623.1616	623.1618	−0.32	C_28_H_32_O_16_	314, 299
M2	7.4	477.1044	477.1039	1.05	C_22_H_22_O_12_	314, 271, 257, 243
M3	8.6	315.0512	315.0510	0.63	C_16_H_12_O_7_	300, 271, 151
M4	3.5	167.0345	167.0350	−2.99	C_8_H_8_O_4_	123
M5	3.4	153.0186	153.0193	−4.57	C_7_H_6_O_4_	109
M6	5.3	151.0394	151.0401	−4.63	C_8_H_8_O_3_	107

### 2.3 Time–Concentration Curve and Sequence of TYP Metabolites

**Figure 3:**
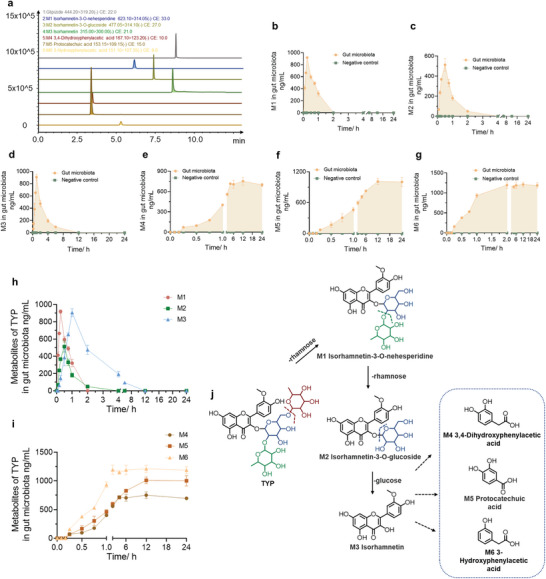
The time–concentration curve of TYP metabolites. a) EIC of TYP metabolites and IS. b) Time‒concentration curves of M1. c) Time‒concentration curves of M2. d) Time‒concentration curves of M3. e) Time‒concentration curves of M4. f) Time‒concentration curves of M5. g) Time‒concentration curves of M6. h) Concentration of three intermediate metabolites at different incubation times. i) Concentration of three ultimate metabolites at different incubation times. j) Metabolic pathway of TYP by the gut microbiota.

To further validate the bacterial metabolites of TYP and explore the production sequence, a standard determination method was established for confirmation through LC‐MS/MS. The retention times of isorhamnetin‐3‐*O*‐neohesperidin, isorhamnetin‐3‐*O*‐glucoside, isorhamnetin, 3,4‐dihydroxyphenylacetic acid, protocatechuic acid, and 3‐hydroxyphenylacetic acid were 6.2, 7.4, 8.6, 3.5, 3.4, and 5.3 min, respectively (**Figure** **3**a). The MRM and retention time are shown in **Table** **2**. The retention times and quantitative ions of the standards were consistent with those of the LC‐Q‐TOF data; therefore, the six bacterial metabolites of TYP were confirmed. In addition, we quantitatively analyzed these metabolites. Among these metabolites, M1–M3 are intermediate bacterial metabolites. M4–M6 were the end products of TYP (Figure 3b–g). The concentrations of M1–M3 first increased with increasing incubation time and then decreased due to complete metabolism (Figure 3b–g). The concentration of M1 reached the highest level after incubating for 15 min, at ≈900 ng mL^−1^ (Figure 3b). Subsequently, the concentration of M1 continued to decrease, indicating that this compound was further metabolized, and the compound was completely metabolized after incubating for 2 h. While the concentration of M2 reached the highest level of ≈500 ng mL^−1^ after incubating for 30 min (Figure 3c). M2 was completely metabolized after incubating for 4 h. The concentration of isorhamnetin reached its highest level after incubating for 1 h and then decreased until it was fully metabolized after 12 h (Figure 3d). The highest concentration of M3 was ≈1 µg mL^−1^. These metabolites were not detected in the negative control group.

**Table 2:** Mass spectrometry parameters of TYP and its metabolites.

Analyte	Precursor ion [*m/z*]	Quantification ion [*m/z*]	*t*_R_ [min]
TYP	769.10	314.10	4.5
3,4‐Dihydroxyphenylacetic acid	167.10	123.20	3.5
Protocatechuic acid	153.15	109.15	3.4
3‐Hydroxy phenylacetic acid	151.10	107.35	5.3
Glipizide (IS)	444.20	319.20	8.8
Isorhamnetin‐3‐*O*‐nehesperidine	623.10	314.05	6.2
Isorhamnetin‐3‐*O*‐glucoside	477.05	314.10	7.4
Isorhamnetin	315.00	300.00	8.6

The other three metabolites, M4–M6 were the end products of TYP. These metabolites were not detected in the negative control group (Figure 3e–g). In the gut microbiota group, these metabolites were detected after 15 min. The concentration of M4 continued to increase after incubating for 15 min to 4 h and reached a plateau at 800 ng mL^−1^ after 2 h (Figure 3e). In contrast to the trend observed for M4, the concentration of M5 plateaued after incubating for 12 h and continued to increase within 15 min to 12 h of incubation (Figure 3f). The concentration of M6 showed a similar change trend to that of M4, in which the concentration continued to increase within 15 min to 2 h of incubation and then reached a plateau after 2 h and remained basically unchanged (Figure 3g).

According to the production curve of these metabolites, the TYP metabolism pathway and the sequence of production of these metabolites could be inferred. The concentrations of M1, M2, and M3 reached their highest levels after incubating for 15 min, 30 min, and 1 h, respectively, and were completely metabolized after 2, 4, and 12 h, respectively (Figure 3h). Therefore, the production of these three metabolites occurred sequentially. M1 was generated by removing one rhamnose from TYP, and then M2 was produced by removing one rhamnose from M1. Furthermore, M3 was produced by removing one glucose from M2. In contrast, the other three phenolic acid metabolites were not formed in an obvious sequence, but all three metabolites began to be generated after incubating for 15 min (Figure 3i); therefore, we speculated that they were metabolites of M1–M3. The pathway of TYP metabolism by the gut microbiota is shown in Figures 3j.

### 2.4 The Metabolism of TYP by Intestinal Bacteria Was Mediated by Rha, Glu, Flr, and Chi

**Figure 4:**
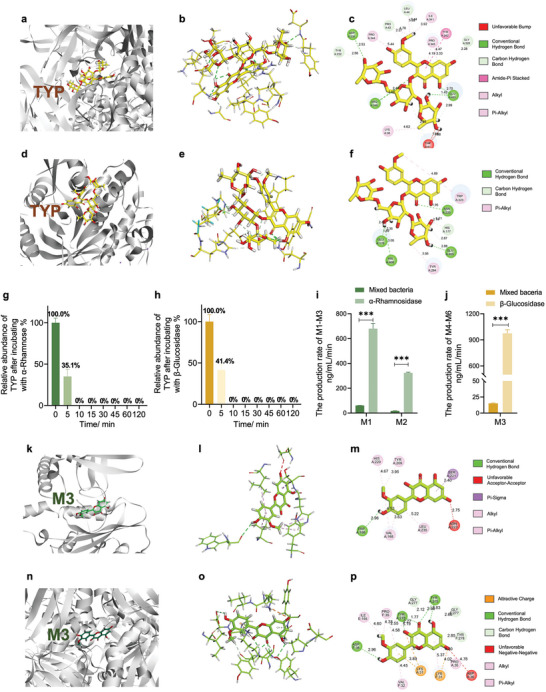
The metabolism of TYP by intestinal bacteria was mediated by Rha and Glu. a) Virtual docking of TYP with Rha (pdb: 6i60) by Libdock. b) Possible interactions between TYP and Rha. c) 2D diagram of possible interactions between TYP and Rha. d) Virtual docking of TYP with Glu by Libdock (pdb: 8wfw). e) Possible interactions between TYP and Glu. f) 2D diagram of possible interactions between TYP and Glu. g) Relative abundance of TYP after incubating with Rha. h) Relative abundance of TYP after incubating with Glu. i) The production rate of M1 and M2 in mixed bacteria and Rha. j) The production rate of M3 in mixed bacteria and Glu. k) Virtual docking of M3 with Flr (pdb: 7d39) by Libdock. l) Possible interactions between TYP and Flr. m) 2D diagram of possible interactions between M3 and Flr. n) Virtual docking of M3 with Chi (pdb: 3zph) by Libdock. o) Possible interactions between M3 and Chi. p) 2D diagram of possible interactions between M3 and Chi. ***p < 0.001.

The enzymes involved in the metabolism of TYP were investigated and included two hydrolytic enzymes and two other enzymes: bacterial α‐rhamnosidase (Rha) and bacterial β‐glucosidase (Glu), flavone reductase (Flr) and chalcone isomerase (Chi). According to metabolic pathway of TYP by the gut microbiota (Figure 3j), Rha and Glu were involved in the transformation of TYP into M1–M3. A specific flavonoid reductase (Flr), which specifically acts on the C2─C3 double bond of the flavonoid C ring, has been found in the gut microbe *Flavonifractor plautii*.^[^ ^37^ ^]^ In addition, Chi was found involved in ring fission reaction of flavonoids.^[^ ^38^ ^]^ Subsequently, the series of enzymes were validated. TYP and Rha (pdb: 6i60) exhibited excellent docking performance, with a LibDock score of 132.724 (**Figure** **4**a–c). In addition, TYP could anchor to the binding site of Rha (pdb: 6i60) through multiple interactions with its scaffold and side chains, including carbon‒hydrogen bonds, conventional hydrogen bonds, π–alkyl interactions, and alkyl interactions. TYP and Glu (pdb: 8wfw) also exhibited excellent docking performance (Figure 4d–f), with a LibDock score of 119.681. Therefore, TYP was metabolized by combining Rha and Glu from the gut microbiota. To further verify the metabolism of TYP, Rha and Glu were incubated with TYP at 37 °C. After incubation with Rha for 5 min, the relative abundance of TYP was 35.1% (Figure 4g). TYP wasmetabolized very quickly, and TYP was completely metabolized within 10 min. Similarly, within 10 min, TYP was completely metabolized by Glu (Figure 4h). Therefore, the metabolism of TYP by intestinal bacteria is mediated by Rha and Glu. Meanwhile, compared with intestinal contents of mice, the production rate of M1 and M2 in Rha group was significantly increased (Figure 4i) and the production rate of M3 in Glu group was significantly increased (Figure 4j). Subsequently, Flr (pdb: 7d39) and Chi (pdb: 3zph) were validated. M3 showed excellent docking performance with Flr and Chi, with LibDock score of 123.547 (Figure 4k–m) and 116.316 (Figure 4n–p), respectively. According to above data, the metabolism of TYP by intestinal bacteria was likely mediated by enzymes Rha, Glu, Flr, and Chi.

### 2.5 TYP Can be Metabolized by *F. Plautii*

**Figure 5:**
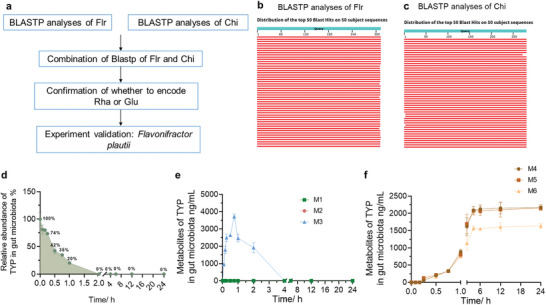
TYP can be metabolized by *F. plautii*. a) The process of searching for strains that may metabolize TYP. b) BLASTp analysis of Flr. c) BLASTp analysis of Chi. d) Relative abundance of TYP after incubating with *F. plautii*. e) M1–M3 levels in *F. plautii*. f) M4–M6 levels in *F. plautii*.

To further verify that the metabolism of TYP is closely related to the gut microbiota, pseudo germ‐free (PGF) ob/ob mice were established through oral antibiotics. Compared with the control group, the anaerobic and aerobic bacterial communities in the PGF group were inhibited by 99% and 89%, respectively (Figure S5a–d, Supporting Information). The intestinal contents of PGF mice were incubated with TYP and the intestinal contents of PGF mice could not metabolize TYP (Figure S6a, Supporting Information). Next, strains that may metabolize TYP were searched (**Figure** **5**a). Due to the large number of strains encoding Rha and Glu, strains containing both Flr and Chi were first searched. A BLASTp analysis on the Flr protein sequence was first performed in the NCBI database (Flr sequence: KGF53654.1^[^ ^37^ ^]^), and the alignment values of the top 50 sequences were all over 98% (Figure 5b). The specific strains are shown in **Table** **3**. Subsequently, a BLASTp analysis on the Chi protein sequence was also performed (Chi sequence: KGF53655.1^[^ ^37^ ^]^), and the alignment values of the top 50 sequences were all over 98% (Figure 5c). The specific strains are shown in **Table** **4**. Tables 3 and 4 were analyzed, and strains containing both Flr and Chi were found, as shown in **Table** **5**. These common strains all encode Glu or Rha. Among them, only *F. placiti* can be purchased. *F. plautii* was incubated with TYP in vitro, and TYP can indeed be rapidly metabolized by *F. plautii* and M3–M6 were detected (Figure 5d–f). In addition, we performed metabolic experiments on TYP in other single bacteria. Some common and easily purchased bacteria which encode Glu or Rha, including *Bacteroides thetaiotaomicron*, *B. uniformis, A. muciniphila*, and *Lactobacillus rhamnosus GG*, were selected. TYP was incubated with these bacteria within 24 h, after which the relative abundance of TYP was determined. These four strains of bacteria were able to rapidly metabolize TYP within an hour (Figure S6b–e, Supporting Information).

**Table 3:** BLASTp analyses of Flr.

Scientific name	Query cover	Accession
*Clostridium sp. ATCC BAA‐442*	100%	ERI73074.1
*Flavonifractor*	100%	WP_035301429.1/ WP_009257602.1
*Flavonifractor plautii*	100%	WP_349145511.1/ WP_195494860.1/ EHM54433.1/ WP_138308064.1/ WP_195640746.1/ WP_039996523.1/ WP_347551470.1/ MDU3678752.1/ WP_347060757.1/ 7D38_A
*Pseudoflavonifractor phocaeensis*	99%	WP_342234152.1
*Oscillospiraceae bacterium*	99%	BDF66441.1/MCC8122698.1
*Clostridiales bacterium*	99%	MBS5521700.1/MCD8149394.1
*Clostridium carboxidivorans*	99%	WP_007061342.1
*Enterocloster citroniae*	99%	MBE7724080.1
*Clostridium scatologenes*	99%	WP_029162284.1
*Clostridium drakei*	99%	WP_032075364.1
*Clostridium sp. JS66*	99%	WP_318739986.1
*Tepidanaerobacter acetatoxydans*	99%	WP_013779276.1
*Eubacterium sp*.	100%	MCR5784334.1/ MCI8993960.1
*Clostridium muellerianum*	99%	WP_169299193.1
*Flexilinea sp*.	99%	MBP8965731.1/HQN61859.1
*Konateibacter massiliensis*	99%	WP_099466635.1
*Clostridium fermenticellae*	99%	WP_119972742.1
*Parasporobacterium paucivorans*	99%	WP_073993363.1
*Clostridium sp*.	99%	MDO5518237.1
*Clostridium beijerinckii*	99%	WP_026888969.1/ MBE6088657.1/ WP_012059238.1
*Clostridium thailandense*	99%	WP_218319593.1
*Bacillota bacterium*	99%	MBP2651857.1
*Clostridium sp. VAP51*	99%	WP_017353480.1
*Clostridium sp. ZBS18*	99%	WP_252242125.1
*Clostridium*	99%	WP_105176054.1/WP_017209245.1
*Deltaproteobacteria bacterium*	99%	MBN1905572.1
*Clostridium sp. CH2*	99%	WP_252235090.1
*unclassified Clostridium*	99%	WP_252223900.1
*Novisyntrophococcus fermenticellae*	98%	WP_230399571.1
*Clostridium psychrophilum*	99%	WP_216290467.1
*Clostridium estertheticum*	99%	WP_216172353.1
*Brotonthovivens ammoniilytica*	99%	WP_158425727.1/WP_216124924.1

**Table 4:** BLASTp analyses of Chi.

Scientific name	Query cover	Accession
*Flavonifractor*	100%	WP_007488562.1
*Flavonifractor plautii*	100%	WP_347060758.1/MDU6201402.1/WP_256291642.1/WP_271906344.1/WP_204681754.1/WP_347551468.1
*uncultured Flavonifractor sp*.	100%	WP_288859233.1
*Oscillospiraceae bacterium*	100%	BDF66442.1/MDD6161080.1/MCR5649220.1/MFG6350675.1/MDE6955050.1/MCI9221944.1/MBR6311960.1/MBR5489699.1/MBE7017300.1/MBR5709020.1/WP_186906407.1
*Pseudoflavonifractor phocaeensis*	100%	WP_342234151.1
*Flexilinea sp*.	100%	HOG61101.1/MBP8965732.1/HNY19177.1/HPG19264.1/HPS47981.1/HRY20297.1
*Agrilactobacillus yilanensis*	100%	WP_125715151.1
*uncultured Flavonifractor sp*.	100%	WP_317325500.1/WP_297233017.1/WP_317324750.1/WP_297196978.1/WP_300167717.1
*Enterococcaceae bacterium*	100%	MCI1902848.1
*Flavonifractor sp. AGMB03687*	100%	WP_209345349.1
*Flavonifractor sp. An82*	100%	WP_087338499.1
*Clostridium sp. SY8519*	100%	WP_013976971.1
*Clostridiales bacterium*	100%	HCO62140.1
*Baileyella intestinalis*	100%	WP_270304070.1
*Mobilibacterium timonense*	100%	WP_077390608.1/WP_273236223.1
*Eubacterium sp*.	100%	MCM1121994.1
*Lachnospiraceae bacterium*	100%	MCQ2512024.1/MCQ2492684.1
*Lawsonibacter sp*.	100%	WP_287879341.1
*Tepidanaerobacter acetatoxydans*	100%	WP_013779275.1
*Clostridium caldaquaticum*	100%	WP_252132092.1
*Clostridium intestinale*	100%	WP_315110818.1/WP_326512771.1
*Thermodesulfobacteriota bacterium*	100%	MFC1890980.1
*Caloramator sp. E03*	100%	WP_138979638.1

**Table 5:** The common BLASTp analyses of Flr and Chi.

Scientific name	Query cover	Whether encoding Rha or Glu
*Flavonifractor*	100%	encoding Glu
*Flavonifractor plautii*	100%	encoding Glu
*Oscillospiraceae bacterium*	99%	encoding Glu and Rha
*Pseudoflavonifractor phocaeensis*	99%	encoding Glu
*Flexilinea sp*.	99%	encoding Glu
*Clostridiales bacterium*	99%	encoding Glu and Rha
*Eubacterium sp*.	99%	encoding Glu and Rha
*Tepidanaerobacter acetatoxydans*	99%	encoding Glu

### 2.6 The Role of Rha and Glu in TYP Metabolism Was Verified by Gene Knockout and Overexpression Techniques

**Figure 6:**
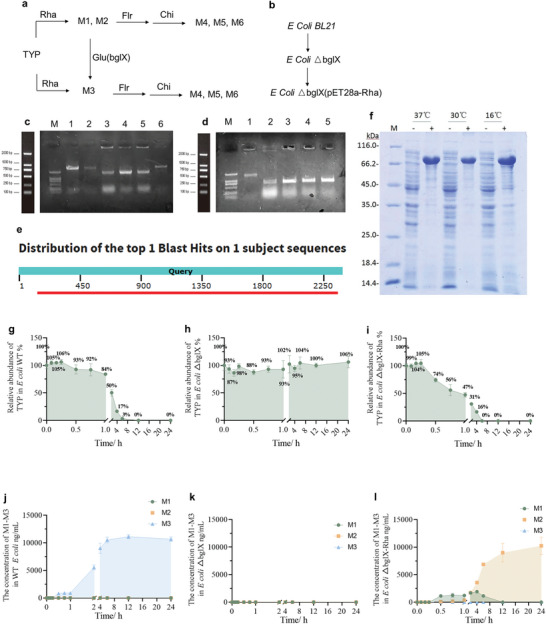
The roles of Glu and Rha in TYP metabolism were verified by gene knockout and overexpression. a) The metabolic process of TYP by Rha, Glu, Flr, and Chi. b) Gene knockout and overexpression process in *E. coli*. c) Gel electrophoresis of products of PCR of knockout bglX gene (bglX gene was replaced by *kan* gene). M: DL2000 DNA Marker. 1: WT‐BL21(DE3) (negative control). 2–6: products of PCR of knockout bglX gene (Clones 1–5). d) Gel electrophoresis of products of elimination *kan* gene amplified by PCR. M: DL2000 DNA Marker. 1: products of PCR of knockout bglX gene (bglX gene was replaced by *kan* gene) (negative control). 2–5: products of elimination kan gene amplified by PCR (Clones 1–4) e) Sequence alignment of plasmid pET28a‐Rha and gene Rha (NZ_CP040530.1). f) Result of SDS‐PAGE analysis of the purified enzyme. m: protein marker. −: no IPTG. +: IPTG. g) Relative abundance of TYP in *E. coli BL21(DE3)* WT. h) Relative abundance of TYP in *E. coli BL21(DE3)* ▵bglX. i) Relative abundance of TYP in *E. coli BL21(DE3)* ▵bglX (pET28a‐Rha). j) The concentration of M1–M3 in *E. coli BL21(DE3)* WT. k) The concentration of M1–M3 in *E. coli BL21(DE3)* ▵bglX. l) The concentration of M1–M3 in *E. coli BL21(DE3)* ▵bglX(pET28a‐Rha).

As shown in **Figure** **6**a, TYP was metabolized by Rha, Glu, Flr, and Chi. The roles of Rha and Glu were further confirmed through gene knockout and overexpression techniques. The glucosidase of *Escherichia coli* is bglX (gene ID: 946682) and the α‐l‐rhamnosidase gene (NZ_CP040530.1) from *B. thetaiotaomicron* was codon optimized for expression in *E. coli BL21(DE3)* ▵bglX, as shown in Figure 6b. Gene bglX was knocked out by λred homologous recombination technology. First, gene bglX was replaced with *kan* gene. Using wild‐type BL21 (DE3) as a negative control, the negative result was 2947 bp, as shown in channel 1 of Figure 6c. The PCR results showed that the resistance *kan* gene successfully replaced the bglX gene, with a length of 1695 bp, as shown in channel 3–5 of Figure 6c. Subsequently, *kan* gene was eliminated. As shown in channel 1 of Figure 6d, products of bglX gene replaced by *kan* gene were used as negative control with a length of 1695 bp. PCR results showed that *kan* gene was successfully eliminated in clones 1–4 with a length of 740 bp, as shown in channel 2–5 of Figure 6d. All clones 1–4 were identified as positive and the genome of clone 1 was performed with PCR sequencing. Sequencing shows that the bglX gene has been successfully eliminated. After successfully constructing the strain of *E. coli BL21(DE3)* ▵bglX, we began the experiment of overexpressing rhamnosidase. After constructing plasmid pET28A‐Rha, PCR sequencing was performed. The sequencing results were compared with the sequence of α‐l‐rhamnosidase gene (NZ_CP040530.1) with a length of 2214 bp, which was 100% consistent with plasmid pET28A‐Rha, as shown in Figure 6e. The expression of Rha was further verified by protein purification experiment. As shown in Figure 6f, the molecular mass of the enzyme was estimated to be 80 kDa. The above results proved the successful construction of the strain *E. coli BL21(DE3)* ▵bglX and *E. coli BL21(DE3)* ▵bglX (pET28a‐Rha). And then we carried out functional verification. *E. coli BL21(DE3)* WT, *E. coli BL21(DE3)* ▵bglX, and *E. coli BL21(DE3)* ▵bglX (pET28a‐Rha) were incubated with TYP, respectively, and then TYP and metabolite were measured. As shown in Figure 6g, TYP was fully metabolized by *E. coli BL21(DE3)* WT. The concentration of M3 increased with the extension of incubation time, and M1 and M2 could not be measured in *E. coli BL21(DE3)* WT, as shown in Figure 6j. When gene bglX was knocked out, the metabolism of TYP was inhibited and M1–M3 could not be determined in *E. coli BL21(DE3)* ▵bglX, as shown in Figure 6h,k. When Rha was overexpressed and Glu was knocked out, TYP was rapidly metabolized by *E. coli BL21(DE3)* ▵bglX (pET28a‐Rha). M3 could not be determined due to knockout of Glu. M1 was detected before M2, but with the extension of incubation time, the end product was only M2 (Figure 6i,l). This further verified that TYP was metabolized by Glu and Rha into M1–M3.

### 2.7 Both TYP and TYP Metabolites Promoted the Production of SCFAs

**Figure 7:**
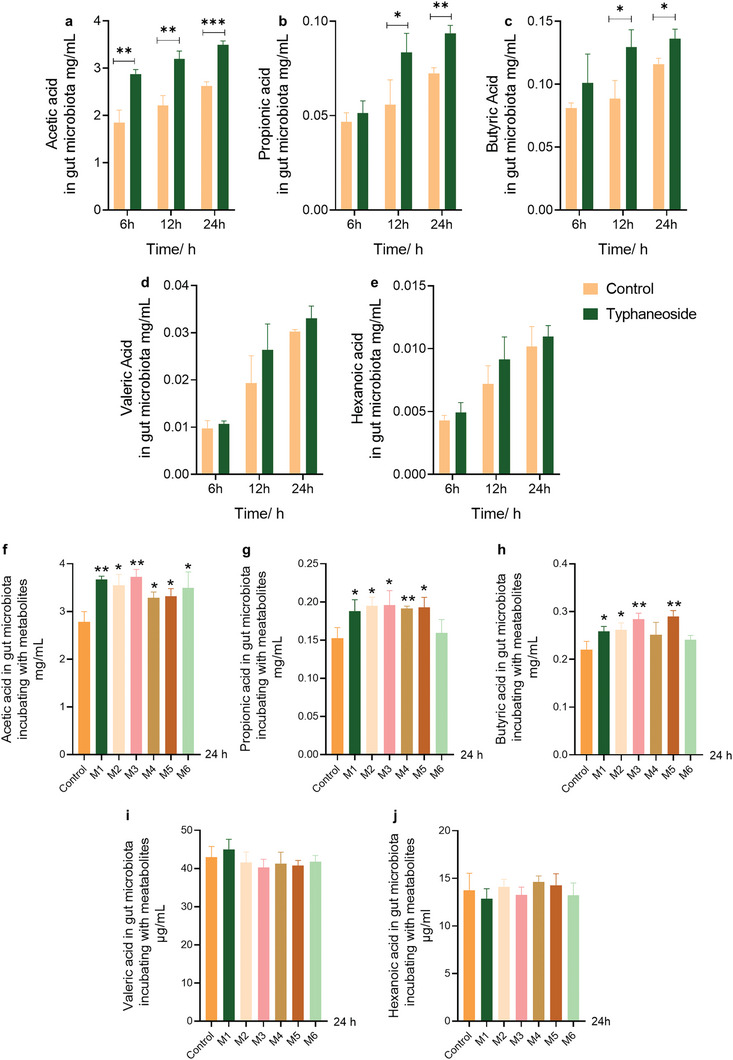
The concentration of SCFAs in the gut microbiota after incubation with TYP and TYP metabolites. a) The concentrations of acetic acid in the gut microbiota after incubating with TYP for 6, 12, and 24 h. b) The concentrations of propionic acid in the gut microbiota after incubating with TYP for 6, 12, and 24 h. c) The concentrations of butyric acid in the gut microbiota after incubating with TYP for 6, 12, and 24 h. d) The concentrations of valeric acid in the gut microbiota after incubating with TYP for 6, 12, and 24 h. e) The concentrations of hexanoic acid in the gut microbiota after incubating with TYP for 6, 12, and 24 h. f) The concentrations of acetic acid in the gut microbiota after incubating with M1–M6 for 6, 12, and 24 h. g) The concentrations of propionic acid in the gut microbiota after incubating with M1–M6 for 6, 12, and 24 h. h) The concentrations of butyric acid in the gut microbiota after incubating with M1–M6 for 6, 12, and 24 h. i) The concentrations of valeric acid in the gut microbiota after incubating with M1–M6 for 6, 12, and 24 h. j) The concentrations of hexanoic acid in the gut microbiota after incubating with M1–M6 for 6, 12, and 24 h. *p < 0.05, **p < 0.01, ***p < 0.001.

Next, the effects of TYP on the gut microbiota were investigated. The concentrations of the most essential bacterial metabolites, SCFAs, were determined after incubating TYP with the gut microbiota for 6, 12, or 24 h. The concentration of SCFAs increased continuously with increasing incubation time (**Figure** **7**a–e). After incubating with TYP for 24 h, the levels of acetic acid, propionic acid, and butyric acid significantly increased (*p* < 0.001, *p* < 0.01, and *p* < 0.05). Among the metabolites, acetic acid level increased most significantly after TYP administration. After incubation with TYP for 6, 12, or 24 h, the concentration of acetic acid increased by 55%, 44%, and 33%, respectively. After varying incubation times, compared with those in the control group, the expression of valeric acid and hexanoic acid was upregulated, but there was no significant difference. These results suggested that TYP can significantly promote the production of SCFAs, including acetic acid, propionic acid, and butyric acid. In addition, to investigate whether the bacterial metabolites of TYP have an effect on the gut microbiota, six metabolites were incubated with the gut microbiota for 24 h. Interestingly, most of the metabolites exhibited effects similar to those of TYP in regulating SCFAs (Figure 7f–j). The three intermediate metabolites significantly promote the generation of acetic acid, propionic acid, and butyric acid, including M1, M2, and M3. Among the three final metabolites, M5 appeared to have the most obvious promotion of acetic acid, propionic acid, and butyric acid production. In summary, TYP and its metabolites promoted the production of SCFAs.

### 2.8 TYP and Some Bacterial Metabolites Can Alter the Lipid Metabolism via Gut Microbiota

**Figure 8:**
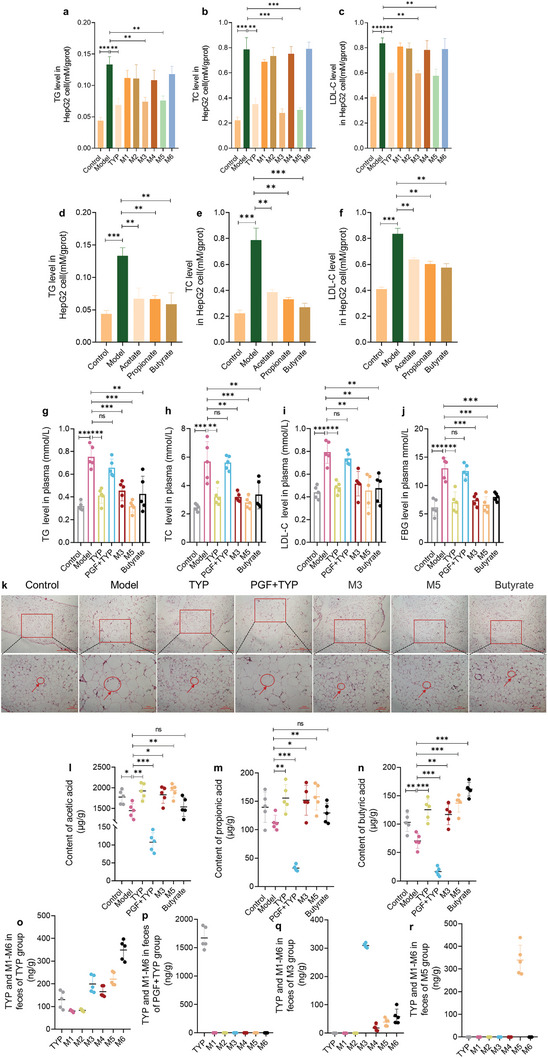
TYP, TYP metabolites, and SCFAs alter lipid metabolism in vitro and in vivo. a) Triglyceride (TG) levels in HepG2 cells after incubating with TYP and M1–M6. b) Total cholesterol (TC) levels in HepG2 cells after incubating with TYP and M1–M6. c) Low‐density lipoprotein cholesterol (LDL‐C) levels in HepG2 cells after incubating with TYP and M1–M6. d) TG levels in HepG2 cells after incubating with SCFAs. e) TC levels in HepG2 cells after incubating with SCFAs. f) LDL‐C levels in HepG2 cells after incubating with SCFAs. g) TG levels in plasma. h) TC levels in plasma. i) LDL‐C levels in plasma. j) Fasting blood glucose (FBG) levels in plasma. k) The staining of epididymal white adipose tissue (eWAT) with H&E. The cell framed in red circles and pointed by red arrows is white fat cell. l) Acetic acid concentrations in feces. m) Propionic acid concentrations in feces. n) Butyric acid concentrations in feces. o) TYP, M1–M6 level in feces of TYP group. p) TYP, M1–M6 level in feces of TYP+PGF group. q) TYP, M1–M6 level in feces of M3 group. r) TYP, M1–M6 level in feces of M5 group. *p < 0.05, **p < 0.01, ***p < 0.001, ns: no significance.

Subsequently, considering the lipid‐lowering activity of *T. angustifolia* L., the effects of TYP on lipid metabolism were investigated. In addition, to verify our hypothesis that the efficacy of TYP is related to its microbial metabolites, the effects of six microbial metabolites and SCFAs on lipid metabolism were explored. A lipid accumulation model was established in HepG2 cells to evaluate the effects of the various treatments. The concentrations of triglycerides (TGs), total cholesterol (TC), and low‐density lipoprotein cholesterol (LDL‐C) were used as indicators of treatment efficacy. The TG, TC, and LDL‐C levels in HepG2 cells increased significantly after modeling, indicating successful establishment of the model (**Figure** **8**a–c). After the administration of TYP and its metabolites, we found that TYP could significantly inhibit the production of TG, TC, and LDL‐C (Figure 8a–c). Among the metabolites, M3 and M5 significantly inhibited TG, TC, and LDL‐C production and improved hyperlipidemia. In addition, the effects of M3 and M5 on TC and LDL‐C were greater than those of TYP. In contrast to the metabolites of TYP, acetate, propionate, and butyrate produced by TYP significantly inhibited TG, TC, and LDL‐C production. Among the three SCFAs, butyrate had the greatest effect (Figure 8d–f). In summary, on the one hand, the lipid‐lowering effects of TYP may be related to its bacterial metabolites M3 and M5; on the other hand, TYP may be related to the promotion of SCFA production, especially that of acetate, propionate, and butyrate.

Next, in vivo experiments were used to verify the efficacy of TYP and its metabolites. In cell experiments, butyrate showed the best efficacy among the SCFAs. Therefore, we investigated the effects of butyrate on hyperlipidemia in vivo. Ob/ob mice are leptin‐deficient mice that are widely used as models for studying obesity and lipid metabolism disorders.^[^ ^39^ ^]^ In our study, ob/ob mice were used as a model, and C57/6J mice were used as a normal control. After 21 d of administration, the plasma TG, TC, LDL‐C, and fasted blood glucose (FBG) levels of ob/ob mice were determined. Compared with those in the control group, the lipid levels, including TG, TC, and LDL‐C levels, in the plasma of ob/ob mice were significantly higher (Figure 8g–j). After 21 d of oral administration, TYP and bacterial metabolites significantly improved hyperlipidemia, and the levels of TC, TG, and LDL‐C were significantly lower. In addition, butyrate significantly reduced the TG, TC, and LDL‐C levels in ob/ob mice (Figure 8g–j). FBG was also measured, and TYP, M3, M5, and butyrate significantly reduced the FBG. Compared with those in the control group, the diameter of epididymal white adipose tissue (eWAT) was greatly elevated in model group, which was offset by TYP and bacterial metabolites (Figure 8k). While, in PGF ob/ob mice, the regulating capacity of TYP in hyperlipidemia was weak. The above results suggested that TYP ameliorated hyperlipidemia by intestinal bacteria. Considering the effect of antibiotics on ob/ob mice was not separately investigated, the disappearance of therapeutic efficacy in the TYP+PGF group may be caused by antibiotics. Therefore, we supplemented corresponding experiments. Ob/ob mice were set as the model group, ob/ob mice were orally administrated antibiotics to be the PGF group. After 21 d of administration, compared with model group, there were no significant differences in TC, TG, LDL, and FBG of PGF group, and the radius of white fat was similar (Figure S7a–f, Supporting Information). Therefore, the weakened efficacy of the TYP+PGF group was due to the elimination of gut microbiota. It was further confirmed that the efficacy of TYP depends on the gut microbiota.

Considering that TYP and its metabolites can significantly promote SCFA production in vitro, SCFA levels were determined in the feces of ob/ob mice 21 d after administration. Compared with those in the control group, the contents of acetic acid and propionic acid in the stool of ob/ob mice decreased, while the content of butyric acid decreased significantly (*p < 0.01) (Figure 8l–n). TYP, M3, and M5 administration increased the contents of SCFAs, especially butyric acid. Meanwhile, it was observed that the use of antibiotics substantially eliminated SCFAs in the feces of each group. In vivo experiments further verified the efficacy of TYP in improving hyperlipidemia, and the efficacy of TYP was related to two important bacterial metabolites, M3 and M5.

In addition, the levels of TYP and M1–M6 in feces after TYP administration were further determined to verify whether M1–M6 are bacterial metabolites (Figure 8o–r; Figure S8a–c, Supporting Information). TYP and M1–M6 can be detected in the feces of the TYP group (Figure 8o). Compared with the TYP group, the fecal TYP content in the TYP+PGF group increased by more than 10 times, and M1–M6 was not detected in the PGF+TYP group (Figure 8p). These data showed that M1–M6 are bacterial metabolites of TYP. In plasma of TYP group, M3–M6 were detected but TYP, M1, and M2 were not detected (Figure S8d, Supporting Information). By comparing the plasma pharmacokinetic curves and pharmacokinetic parameters of TYP, M3, and M5 (Figures S2a,b and S9a–d, Supporting Information; **Tables** 6, 7, 8, 9, 10, 11), M3 and M5 showed higher bioavailability (TYP bioavailability: 2.78%, M3 bioavailability: 9.37%, M5 bioavailability: 9.00%). Therefore, TYP may exert its anti‐hyperlipidemia effects through metabolized into M3 and M5, which showed higher bioavailability.

**Table 6:** Pharmacokinetic parameters of TYP in C57BL/6 mice after oral administration of TYP (100 mg kg^−1^).

Parameters	Unit	Mean	SD
AUC(0‐t)	µg L^−1^ min^−1^	70219.59	8707.619
AUC(0‐∞)	µg L^−1^ min^−1^	76607.546	7782.836
*C*_max_	µg L^−1^	2688.015	951.547

**Table 7:** Pharmacokinetic parameters of TYP in C57BL/6 mice after intravenous administration of TYP (5 mg kg^−1^).

Parameters	Unit	Mean	SD
AUC(0‐t)	µg L^−1^ min^−1^	131126.195	11127.31
AUC(0‐∞)	µg L^−1^ min^−1^	137931.052	16043.681
*C*_max_	µg L^−1^	7700.973	648.066

**Table 8:** Pharmacokinetic parameters of M3 in C57BL/6 mice after oral administration of M3 (100 mg kg^−1^).

Parameters	Unit	Mean	SD
AUC(0‐t)	µg L^−1^ min^−1^	226874.48	37501.78
AUC(0‐∞)	µg L^−1^ min^−1^	294575.858	66256.265
*C*_max_	µg L^−1^	2991.089	1162.454

**Table 9:** Pharmacokinetic parameters of M3 in C57BL/6 mice after intravenous administration of M3 (5 mg kg^−1^).

Parameters	Unit	Mean	SD
AUC(0‐t)	µg L^−1^ min^−1^	155930.438	61709.355
AUC(0‐∞)	µg L^−1^ min^−1^	157108.968	61712.607
*C*_max_	µg L^−1^	7333.341	1851.811

**Table 10:** Pharmacokinetic parameters of M5 in C57BL/6 mice after oral administration of M5 (100 mg kg^−1^).

Parameters	Unit	Mean	SD
AUC(0‐t)	µg L^−1^ min^−1^	284583.233	47175.404
AUC(0‐∞)	µg L^−1^ min^−1^	285882.95	45657.945
*C*_max_	µg L^−1^	10038.369	3005.098

**Table 11:** Pharmacokinetic parameters of M5 in C57BL/6 mice after intravenous administration of M5 (5 mg kg^−1^).

Parameters	Unit	Mean	SD
AUC(0‐t)	µg L^−1^ min^−1^	153402.985	74895.329
AUC(0‐∞)	µg L^−1^ min^−1^	158667.281	79920.42
*C*_max_	µg L^−1^	8541.143	3094.138

### 2.9 TYP Can Improve Intestinal Barrier and Adjust Intestinal Microbiota Composition

**Figure 9:**
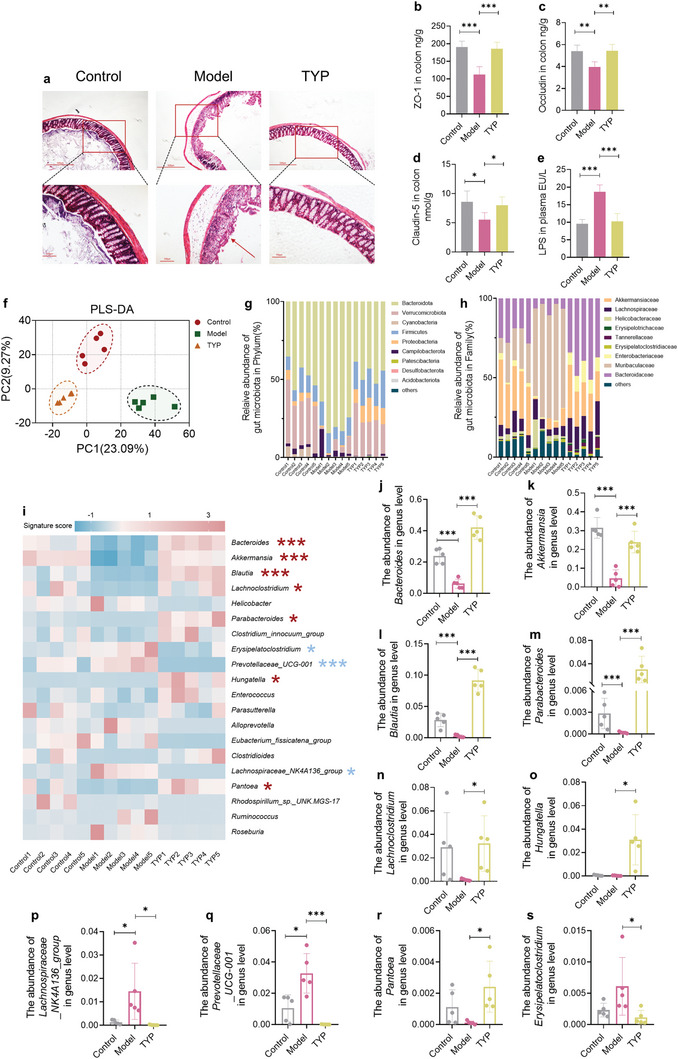
TYP can improve intestinal barrier and adjust intestinal microbiota composition. a) Histological H&E section of the colon. b) The concentration of ZO‐1 in colon. c) The concentration of Occludin in colon. d)The concentration of Claudin‐5 in colon. e) The concentration of LPS in plasma. f) β‐diversity analysis of fecal samples. g) Relative abundance of gut microbiota in Phylum. h) Relative abundance of gut microbiota in Family. i) Top 20 heatmap of the gut microbiota in genus level. j) The abundance of *Bacteroides* in genus level. k) The abundance of *Akkermansia* in genus level. l) The abundance of *Blautia* in genus level. m) The abundance of *Parabacteroides* in genus level. n) The abundance of *Lachnoclostridium* in genus level. o) The abundance of *Hungatella* in genus level. p) The abundance of *Lachnospiraceae_NK4A136_group* in genus level. q) The abundance of *Prevotellaceae_UCG‐001* in genus level. r) The abundance of *Pantoea* in genus level. s) The abundance of *Erysipelatoclostridium* in genus level. *p < 0.05, **p < 0.01, ***p < 0.001.

Subsequently, since intestinal bacteria play an important role in the efficacy of TYP, we further analyzed the effects of TYP on intestinal barrier and intestinal microbiota. Compared with control group, inflammatory injury was observed in colon of model group, but the inflammatory injury disappeared after TYP administration (Figure 9a). Meanwhile, indicators related to the intestinal barrier were measured, including ZO‐1, Occludin, Claudin‐5, and LPS. Destruction of the intestinal barrier was observed in model group and TYP could inhibit the destruction of the intestinal barrier (**Figure** **9**b–e). After 21 d of TYP oral administration, fecal samples of each animal group were analyzed by 16S rRNA gene. β‐diversity analysis showed significant clustering and separation between groups, with significant altered fecal microbiota between the control and model groups, which TYP appeared to reverse this trend (Figure 9f). In Phylum level, relative abundance analysis revealed that bacteria from the phylum Verrucomicrobia were enriched in TYP group (Figure 9g). At the family level, model group led to a reduction in the abundance of Akkermansiaceae but the family Akkermansiaceae were enriched in TYP group (Figure 9h). At the genus level, TYP group led to an increase in the abundance of *Bacteroides*, *Akkermansia, Blautia*, *Parabacteroides*, and *Lachnoclostridium* and a reduction in the abundance of *Erysipelatoclostridium*. Overall, the above data showed that TYP could restore gut microbiota homeostasis and specifically regulate *Bacteroides* and *Akkermansia* abundance (Figure 9i–s).

### 2.10 TYP Can Regulate SCFA Producing Strains at the Species Level and Promote Butyrate Metabolism

**Figure 10:**
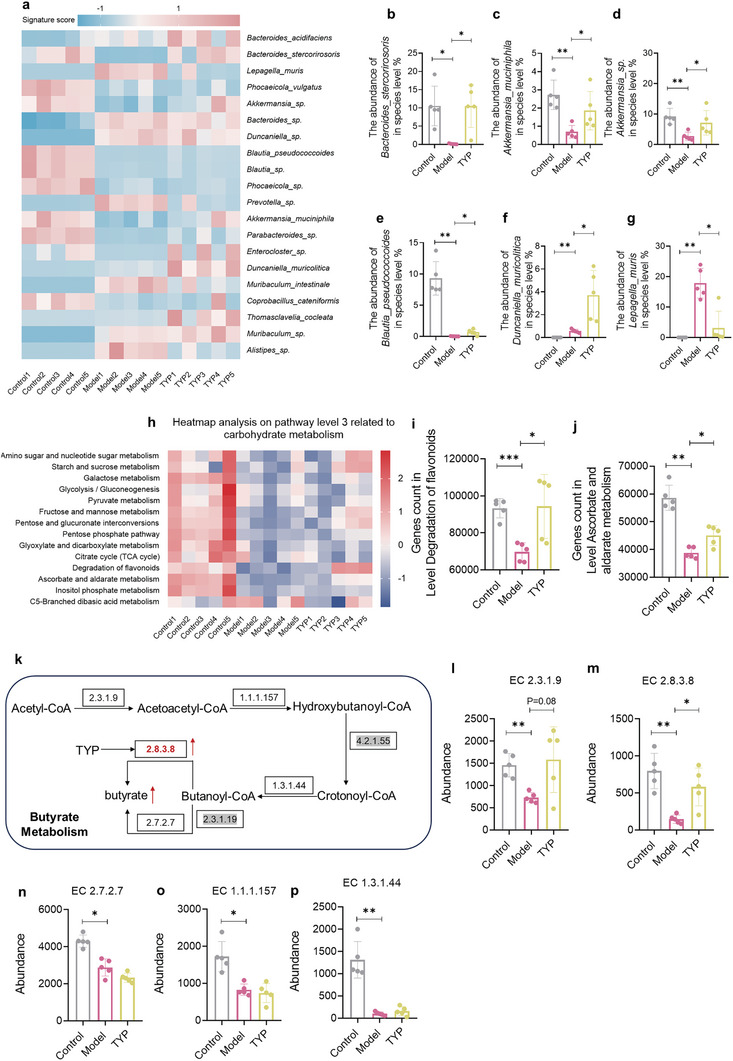
The metagenome analysis of feces after TYP administration. a) Top 20 heatmap of the gut microbiota in species level. b) The abundance of *Bacteroides_stercorirosoris* in species level. c) The abundance of *Akkermansia_muciniphila* in species level. d) The abundance of *Akkermansia_sp*. in species level e) The abundance of *Blautia_pseudococcoides* in species level. f) The abundance of *Duncaniella_muricolitica* in species level. g) The abundance of *Lepagella_muris* in species level. h) Heatmap analysis on KEGG pathway level 3 related to carbohydrate metabolism. i) Genes count in level: Degradation of flavonoids. j) Genes count in level: Ascorbate and aldarate metabolism. k) Butyrate metabolism pathway (The enzyme labeled with gray was not detected in the metagenome analysis). l) Abundance of EC 2.3.1.9. m) Abundance of EC 2.8.3.8. n) Abundance of EC 2.7.2.7. o) Abundance of EC1.1.1.157. p) Abundance of EC 1.3.1.44. *p < 0.05, **p < 0.01, ***p < 0.001.

Subsequently, metagenomic analysis was conducted and the results showed that TYP increased the abundance of SCFA‐producing strains at the species level, including Bacteroides_stercorirosoris,^[^ ^40^ ^]^ Akkermansia_sp,^[^ ^41^ ^]^ Blautia_pseudococcoides,^[^ ^42^ ^]^ and Akkermansia_muciniphila^[^ ^41^ ^]^ (**Figure** **10**a–e). The other two strains at the species level: Duncaniella_muricolitica and Lepagella_muris, which were different between groups, have been less studied (Figure 10f,g). Based on the KEGG metabolic pathway, pathways related to carbohydrate metabolism were analyzed (Figure 10h–j). Compared with the Control group, the flavonoid metabolism pathway in the model group was significantly inhibited, and TYP could significantly upregulate the flavonoid metabolism pathway (Figure 10i). In addition, metagenomic analysis showed that TYP can regulate butyrate metabolism. Butyrate is produced by Acetyl‐CoA through a series of metabolic pathways (Figure 10k), of which the core step is butyryl CoA produces butyrate. Pathway 1 is butyryl CoA produces butyrate through butyric kinase (EC 2.7.2.7), and pathway 2 is butyryl CoA produces butyric acid through butyryl CoA transferase (EC 2.8.3.8). Compared with control group, a series of enzymes that promote butyrate production were inhibited in model group (Figure 10l–p). TYP can promote the production of butyrate by only promoting butyryl CoA transferase (EC 2.8.3.8) (Figure 10m). The above data of metagenomic analysis could explain the promoting effect of TYP on butyrate.

### 2.11 Fecal Microbiota Transplantation Improved Hyperlipidemia and the Metabolite Combination of TYP Showed Excellent Efficacy

**Figure 11:**
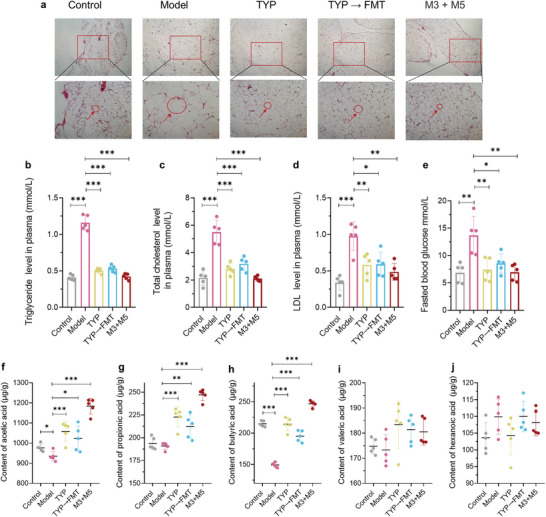
FMT improved hyperlipidemia and the metabolite combination of TYP showed more obvious efficacy. a) The staining of epididymal white adipose tissue (eWAT) with H&E. The cell framed in red circles and pointed by red arrows is white fat cell. b) TG levels in plasma. c) TC levels in plasma. d) LDL‐C levels in plasma. e) FBG levels in plasma. f) Acetic acid concentrations in feces. g) Propionic acid concentrations in feces. h) Butyric acid concentrations in feces. i) Valproic acid concentrations in feces. j) Hexanoic acid concentrations in feces. *p < 0.05, **p < 0.01, ***p < 0.001, ns: no significance.

Our previous experiments proved that TYP can adjust the structure of intestinal microbiota, and TYP metabolites M3 and M5 can effectively improve hyperlipidemia. To further verify the role of intestinal microbiota and metabolites, we conducted a fecal microbiota transplantation (FMT) experiment. We transplanted feces of mice after TYP administration into ob/ob mice and added a group of M3 and M5 combination experiments. The results of FMT experiment showed that after transplantation of feces after TYP administration into ob/ob mice, the hyperlipidemia of ob/ob mice was significantly improved, the diameter of white fat was significantly reduced, as shown in **Figure** **11**a. And plasma indexes including TG, TC, LDL‐C, and FBG were significantly decreased, as shown in Figure 11b–e. These results further confirmed that improvement of TYP on hyperlipidemia is mediated by intestinal microbiota. The combination of M3 and M5 also showed therapeutic effect on improving hyperlipidemia. After the combination of M3 and M5, the reduction in white fat diameter was better than that of TYP, as shown in Figure 11a. Meanwhile, the degree of reduction in plasma efficacy indicators such as TG, TC, LDL‐C and FBG was better than that of TYP, as shown in Figure 11b–e. SCFA in the feces of each group was determined, and similar to the previous results, acetic acid, propionic acid and butyric acid were significantly regulated, as shown in Figure 11f–j. After transplantation of feces after TYP administration into ob/ob mice, acetic acid, propionic acid, and butyric acid in feces were significantly increased, but the contents of acetic acid, propionic acid, and butyric acid in FMT group were lower than those in TYP group, as shown in Figure 11f–h. It is worth noting that after the combination of M3 and M5, the contents of acetic acid, propionic acid, and butyric acid increased significantly, and the contents were higher than those of TYP group, as shown in Figures 11f‐j  .

## 3 Discussion

Cattail pollen is the dried pollen of *T. angustifolia* L. Cattail pollen is widely used in a variety of prescriptions, including Shixiao Powder, Wenshen Xiaozheng Tang, and Ruanjian Qingmai Granules. According to the 2020 edition of Chinese Pharmacopoeia, TYP is a quality control component of cattail pollen, and several literature reports that TYP is an important substance in the formula in which TYP participates.^[^ ^3^, ^5^, ^6^, ^43^, ^44^, ^45^ ^]^ TYP has been found to have a variety of pharmacological activities in modern pharmacological studies, including colitis,^[^ ^9^ ^]^ antioxidant,^[^ ^8^ ^]^ liver protection,^[^ ^12^ ^]^ and neuroprotection.^[^ ^10^ ^]^ Therefore, elucidating the mechanism of TYP is not only beneficial to the further study of TYP, but also to the further study of cattail pollen and the formula containing cattail pollen. As a flavonoid structure, TYP has a low bioavailability, which is inconsistent with its pharmacological activity, so the pharmacological activity of TYP may be related to gut microbiota. In this study, from the perspective of gut microbiota and metabolism, we studied the interaction system between TYP and gut microbiota.

During the conversion of TYP to M1–M3, several hydrolysis reactions occurred, including the hydrolysis of rhamnose and glucoside. The distribution of glycosidic bond hydrolases in the gut microbiota is very extensive.^[^ ^46^ ^]^ Through the in vitro metabolism experiment of TYP by single bacteria, especially *B. thetaiotaomicron and B. uniformis*, were speculated to be involved in the metabolism of TYP. Researchers have identified rhamnosidase BT3686 from *B. polyformis*.^[^ ^47^ ^]^ The C‐ring of TYP contains double bonds, and the gut microbiota, where the reduction reaction is most likely to occur, is mostly anaerobic. There may be other undetected metabolites, such as dihydrotyphaneoside and dihydroisothamnetin. The reduction of double bonds may occur quickly, and these undetected metabolites may quickly undergo other reactions and thus preventing them from being detected. M4–M6 were formed by the B ring and part of the C ring of flavonoids, and the main reactions were cleavage and reduction reactions.

A specific flavonoid reductase (Flr), which was found in the gut microbe *F. plautii*, is a key initiation step in the biotransformation of flavonoids by the gut microbiota. Flr can specifically act on the C2─C3 double bond of the flavonoid C ring.^[^ ^37^ ^]^ Interestingly, *B. fragilis* was also found to be involved in cleavage of the flavonoid C ring.^[^ ^48^ ^]^ Chalcone isomerase (Chi) responsible for the downstream reaction steps in flavone metabolism, have been identified.^[^ ^37^ ^]^ Through molecular docking analysis, we verified the bacteria enzymes in the microbial metabolism process of TYP, including Rha, Glu, Flr, and Chi. We further confirmed that the metabolism of TYP is mediated by Rha and Glu through pure enzyme experiments. As for other enzymes Flr and Chi in the metabolic process, we did not explore them further due to the difficulty in obtaining pure enzymes of Flr and Chi.

For the selection of dosage of TYP and metabolites in animal experiments, previous studies showed that the effective dose of sodium butyrate was 100 mg kg^−1^.^[^ ^49^ ^]^ In order to compare the efficacy of each group, the dose was uniformly designed to be 100 mg kg^−1^. Our study is the first to identify the anti‐hyperlipidemia effects of TYP and the metabolites M3 and M5. Although some studies have shown that plant extracts or traditional Chinese patent medicines containing TYP or metabolites have the effect of anti‐hyperlipidemia, such as *Persea americana Mill*,^[^ ^50^ ^]^ *Bidens bipinnata* L.^[^ ^51^ ^]^ containing M5, Bushao Tiaozhi Capsule,^[^ ^52^ ^]^ sea buckthorn flavonoids,^[^ ^53^ ^]^ and Zhixiong Capsule^[^ ^54^ ^]^ containing M3, Shixiao San^[^ ^55^ ^]^ containing TYP. We found for the first time that TYP monomer and metabolite have anti‐hyperlipidemia effect. TYP and metabolites M3 and M5 belong to flavonoids and phenolic acids, respectively. Research has shown that apigenin^[^ ^56^ ^]^ and scutellarin,^[^ ^57^ ^]^ which have structures similar to TYP and M3. And caffeic acid^[^ ^58^ ^]^ and ferulic acid,^[^ ^59^ ^]^ which have structures similar to M5, have therapeutic effects on hyperlipidemia. The clinical dosage of Pushen capsules is 3 g d^−1^ (Approval number of China Food and Drug Administration: Z20040074^[^ ^60^ ^]^). After our calculation and confirmation, the content of TYP in Pushen capsules is 1.3%. If an adult takes PuShen capsules according to clinical dosage, then the daily intake of TYP is 39 mg. According to our experimental dosage (100 mg kg^−1^), it is equivalent to an adult (60 kg) taking 487 mg of TYP, assuming a 12.3 conversion factor. The dose converted from the experiment differs by 12.5 times from the clinical dose. After our calculation and confirmation, the content of TYP in Cattail Pollen reference crude drug was 2.52%. If the same dose of Pushen capsule is converted to Cattail Pollen reference crude drug, then the TYP obtained by an adult from Cattail Pollen reference crude drug is 76 mg, which is 6.4 times different from the dose converted by our experiment. Although the dosage we used was not completely consistent with the clinical dosage, our experimental results indicated that the efficacy of TYP on hyperlipidemia is certain. According to our experimental results, increasing the content of TYP in the preparation may contribute to the efficacy of related preparations in the treatment of hyperlipidemia, and future studies on TYP‐related preparations can refer to our experimental results.

The metabolites of TYP also have extensive pharmacological activities. Isorhamnetin‐3‐*O*‐neohesperidin and isorhamnetin‐3‐*O*‐glucoside have potential hypoglycemic, hypolipidemic, and anti‐inflammatory effects^[^ ^61^, ^62^ ^]^ and can induce the apoptosis of human colon cancer cells through mitochondrial damage.^[^ ^63^ ^]^ M3 has a wide range of pharmacological activities, including anti‐inflammatory,^[^ ^64^ ^]^ anticancer,^[^ ^65^ ^]^ antioxidant, and hypoglycemic effects.^[^ ^66^ ^]^ Like for TYP, a number of studies have shown that M3 also has liver‐protective effects.^[^ ^67^, ^68^, ^69^ ^]^ M3 can target NLRP3/NF‐κB/Nrf2 to combat acetaminophen‐induced hepatotoxicity,^[^ ^69^ ^]^ alleviate CCL4‐induced liver fibrosis in mice by inhibiting AKt‐mediated hepatocyte apoptosis,^[^ ^68^ ^]^ and reduce LPS‐induced acute lung injury by inhibiting mTOR signaling.^[^ ^70^ ^]^ The final products M4–M6 also have many pharmacological activities and are widely studied natural phenolic acids. M5 has antioxidant, anti‐inflammatory and anticancer activities; can regulate metabolic diseases; and has organ protection effects, including protection of the liver and kidney.^[^ ^71^ ^]^ Compared with that of M5, the activity of 3‐hydroxyphenylacetic acid is mainly focused on liver protection and myocardial protection. A number of studies have shown that 3‐hydroxyphenylacetic acid, which is a bacterial metabolite, can enhance the activity of total aldehyde dehydrogenase to protect liver cells.^[^ ^72^, ^73^ ^]^ 3‐Hydroxyphenylacetic acid can alleviate myocardial infarction by regulating the NRF2 pathway and was also identified as a biomarker to distinguish this disease by metabolomics; the level of 3‐hydroxyphenylacetic acid was significantly reduced in stool samples from patients with colorectal cancer.^[^ ^74^ ^]^ In addition, the 3‐hydroxyphenylacetic acid level was also significantly reduced in urine samples from elderly stroke patients with depression.^[^ ^75^ ^]^ 3,4‐Dihydroxyphenylacetic acid can inhibit type 2 diabetes by improving the intestinal barrier integrity.^[^ ^76^ ^]^ Therefore, the bacterial metabolites of TYP are also associated with its pharmacological activity.

Through 16S rRNA gene analysis of feces, it was found that TYP can upregulate the abundance of some bacteria genera, including *Bacteroides, Akkermansia, Blautia*, and *Parabacteroides*. These bacterial genera are all SCFA‐producing bacteria. *A. muciniphila* and *Bacteroides* spp. have been reported to have cholesterol‐lowering functions.^[^ ^77^ ^]^ *B. vulgatus* was also found to ameliorates lipid metabolic disorders.^[^ ^78^ ^]^ Lipid metabolism disorders are the basis of many metabolic diseases, which are closely related to obesity and atherosclerotic diseases. *Parabacteroides merdae* protects against obesity‐associated atherosclerosis by promoting the metabolism of branched chain amino acids into SCFAs. The metabolic process is mediated by porA gene expressed in *P. merdae*.^[^ ^79^ ^]^ Similarly, *Blautia producta* was also found to have pharmacological effects in improving hyperlipidemia.^[^ ^80^ ^]^ Therefore, the lipid‐lowering effect of TYP may be related to these bacterial genera. TYP can downregulate the abundance of *Erysipelatoclostridium*, which appears to be more prevalent in inflammatory bowel disease^[^ ^81^ ^]^ and obesity.^[^ ^82^ ^]^ In addition, the single bacterial experiment results indicated that the metabolic rate of TYP by *B. thetaiotaomicron* and *B. uniformis* is much higher than that of mixed bacteria. Therefore, Bacteroides are the main genus responsible for TYP metabolism.

In our study, we revealed the metabolic pathway of TYP by examining the gut microbiota and quantitatively analyzed the levels of metabolites. The extensive activity of TYP was found to be closely related to the gut microbiota. First, TYP was metabolized by the gut microbiota to produce active bacterial metabolites. The metabolism of TYP by intestinal bacteria is mediated by Rha, Glu, Flr, and Chi. Some of the bacterial metabolites of TYP, such as M3 and M5, played a synergistic role with TYP in altering lipid metabolism. Moreover, TYP and its metabolites promoted the generation of beneficial SCFAs, which were involved in altering lipid metabolism. Therefore, the lipid‐lowering effect of TYP is related to its interaction with the gut microbiota, bacterial metabolites, and SCFAs, the production of which TYP promotes. Finally, the regulatory effects of TYP, bacterial metabolites, and butyrate on hyperlipidemia were verified in vivo. Overall, our research provides new insights into the mechanism of the pharmacological activities of TYP. Our work exemplarily studied TYP and could open avenues to investigate analogous natural products.

## 4 Conclusion

TYP was metabolized into six bacterial metabolites: M1–M6. The bacterial enzymes involved in TYP metabolism were identified, including Rha, Glu, Flr, and Chi. TYP and TYP metabolites significantly promoted the generation of SCFAs. In vivo experiments proved that the two metabolites of TYP, M3, and M5, ameliorated hyperlipidemia, and TYP restored the intestinal barrier and altered the composition of intestinal microbiota. Therefore, the interaction between TYP and gut microbiota drives the improvement of hyperlipidemia.

## 5 Experimental Section

### Materials

TYP, isorhamnetin‐3‐*O*‐neohesperidin, isorhamnetin‐3‐*O*‐glucoside, isorhamnetin, 3,4‐dihydroxyphenylacetic acid, protocatechuic acid, 3‐hydroxyphenylacetic acid, glipizide, acetic acid, propionic acid, butyric acid, valeric acid, and hexanoic acid were purchased from Beijing Solarbio Science & Technology Co., Ltd. The purities of all the standards exceeded 98%. Acetonitrile (HPLC grade) and acetic acid (100%) were purchased from Thermo Fisher Scientific Co., Ltd. Deionized water was purchased from Hangzhou Wahaha Co., Ltd. Anaerobic medium was purchased from Qingdao Haibo Co., Ltd. Luria‐Bertani (LB) medium was purchased from Beijing Solarbio Science & Technology Co., Ltd. HepG2 cells were purchased from Beyotime Biotechnology Co., Ltd. DMEM (with phenol red, with Ca^2+^ and Mg^2+^) was purchased from Procell Life Science & Technology Co., Ltd. Palmitate acid and oleic acid were purchased from Beijing Solarbio Science & Technology Co., Ltd. A TG determination kit, TC test kit, and LDL‐C test kit were obtained from Nanjing Jiancheng Bioengineering Institute Co., Ltd. FBG detection kits were obtained from Beijing Solarbio Science & Technology Co., Ltd. *F. plautii, B. thetaiotaomicron, B. uniformis, A. muciniphila*, and *L. rhamnosus GG* were obtained from Bena Culture Collection Co., Ltd. α‐Rhamnosidase was obtained from Zancheng (Tianjin) Technology Co., Ltd. β‐glucosidase was obtained from Shanghai Macklin Biochemical Technology Co., Ltd. Artificial gastric juice and simulated intestinal fluid were purchased from Beijing Solarbio Science & Technology Co., Ltd. ZO‐1 Elisa kit, Occludin Elisa kit and Claudin‐5 Elisa kit were obtained from Crgent Biotech Co., Ltd. LPS Elisa kit was obtained from Nanjing Jiancheng Bioengineering Institute Co., Ltd.

### Instruments

An LC‐MS/MS8060, LC‐Q‐TOF systems, GC‐2014 (Shimadzu, Japan), constant temperature culture shaker (LYZ‐100, Shanghai Longyue Co., Ltd., China), small table high‐speed centrifuge (Eppendorf 5418, Germany), and analytical balance (XS1050U, METTLER Toledo, Switzerland) were used.

### Animals

C57/6J mice, weighing 18–22 g, were purchased from Sibeifu (Beijing) Biotechnology Co., Ltd. Ob/ob mice, weighing 40–50 g, were purchased from Sibeifu (Beijing) Biotechnology Co., Ltd. All animals had free access to food and water. The temperature was maintained at 22–24 °C, there was a 12 h light/dark cycle, and the relative humidity was set between 40% and 60%. This study complied with the Institutional Guidelines and Ethics of the Chinese Academy of Medical Sciences and Peking Union Medical College and the Committee on Animal Care and Use in Laboratory Institutions (No. 00004376).

### Fingerprint Spectra of Cattail Pollen Reference Crude Drug and Pushen Capsules

0.5 g of Cattail Pollen reference crude drug was taken and weighed accurately. The mixture was placed in a conical flask with a stopper and 200 mL methanol was added accurately. The mixture was weighed accurately. The mixture was cooled for 12 h and then was heated to reflux for 1 h, the mixture was weighed again after cooling and the lost weight was made up with methanol. The mixture was shaken well and filtered, the filtrate was centrifuged at 4 °C and 13 000 rpm for 10 min, after which 100 µL of the supernatant was collected for LC determination.

As for Pushen capsules, 0.5 g of Pushen capsules was weighed accurately and placed in a conical flask with a stopper, 200 mL methanol was added accurately and the mixture was weighed. The mixture was cooled for 12 h and was heated to reflux for 1 h. The mixture was weighed again after cooling, the lost weight was made up with methanol. The mixture was shaken well and filtered, the filtrate was centrifuged at 4 °C and 13 000 rpm for 10 min, after which 100 µL of the supernatant was collected for LC determination.

The chromatographic conditions were as follows: mobile phase A consisted of 0.05% phosphoric acid solution, and mobile phase B consisted of acetonitrile. Chromatographic analysis was performed on an Alltima C18 (4.6 × 250 mm, 5 µm) column at 40 °C, and the flow rate was set to 0.8 mL min^−1^. The gradient elution conditions were as follows: 0.01–5 min, 15% B; 5.01–30 min, 15%–25% B; 30.01–55 min, 25%−95% B; 55.01–63 min, 95%−15% B; and 63.01–70 min, 15% B. The detection wavelength is 254 nm.

### In Vitro Incubation of TYP and the Gut Microbiota

The experiment was divided into two groups: the gut microbiota group and the negative control group. First, the anaerobic medium was prepared according to the manufacturer's instructions, sterilized, and cooled for use. The intestinal contents of six ob/ob mice were mixed, weighed, and added to anaerobic medium at a ratio of 1:20 (w:v); this mixture is referred to as the bacterial solution in the following text. After mixing, funnel and filter paper were used to perform filtration. And the filtrate was divided into two parts. The first part was incubated at 37 °C and 150 rpm for 30 min for the gut microbiota group. The other portion was boiled three times for the negative control group. Under nitrogen flow, the bacterial solution was added to an EP tube, which was pretreated with 10 µL of TYP solution (1 mg mL^−1^). After the bacterial solution was added, the EP tube was immediately covered, sealed with sealing film and placed in a shaker for incubation at 37 °C. The other procedures for the two groups were consistent except for the different bacterial solutions added. A total of three incubation experiments were conducted. The first set of incubation times were 0, 0.5, 1, 2, 4, 6, 12, and 24 h. The second set of incubation times were 0, 5, 10, 15, 30, 45, 60, 90, and 120 min. The third set of incubation times were 0, 5 min, 10 min, 15 min, 30 min, 45 min, 1 h, 2 h, 4 h, 6 h, 12 h, and 24 h.

### In Vitro Incubation of TYP and Artificial Gastric Juice or Simulated Intestinal Fluid

For artificial gastric juice: 10 µL TYP solution (1 mg mL^−1^) was added into EP tubes, then 990 µL artificial gastric juice was added. The mixture solution was incubated at 37 °C. The incubation times were 0, 0.5, 1, 2, 4, 6, 12, and 24 h.

For simulated intestinal fluid: 10 µL TYP solution (1 mg mL^−1^) was added into EP tubes, then 990 µL simulated intestinal fluid was added. The mixture solution was incubated at 37 °C. The incubation times were 0, 0.5, 1, 2, 4, 6, 12, and 24 h.

### Determination of the Levels of TYP and TYP Metabolites by LC‐MS/MS

The chromatographic conditions were as follows: mobile phase A consisted of 0.1% acetic acid, and mobile phase B consisted of acetonitrile. Chromatographic analysis was performed on an Agilent Proshell 120 EC‐C18 (4.6 × 100 mm, 2.7 µm) column at 40 °C, and the flow rate was set to 0.4 mL min^−1^, with glipizide serving as the internal standard. The gradient elution conditions were as follows: 0.01–3 min, 20% B; 3.01–5 min, 20%−45% B; 5.01‐6 min, 45%–95% B; 6.01–9 min, 95% B; and 9.01–13 min, 20% B.

For mass spectrometry, negative‐ion mode was used, and the quantitative ions of TYP and its metabolites are shown in Table 2.

Sample preparation was performed as follows: the internal standard solution was prepared with methanol, and the concentration was 100 ng mL^−1^. The sample prepared in the “In vitro incubation of TYP and gut microbiota” section was added to the methanol internal standard solution in triplicate. After mixing, the mixture was centrifuged at 4 °C and 13 000 rpm for 10 min, after which 100 µL of the supernatant was collected for LC‐MS/MS determination of the levels of TYP and its metabolites.

### Identification of TYP Metabolites by LC‐Q‐TOF

The chromatographic conditions used were the same as those used in the “Determination of TYP and TYP metabolites” section, and the mass spectrometry conditions were as follows: the primary data acquisition range of mass spectrometry was set from *m/z* 50 to *m/z* 1000, and negative‐ion mode was chosen. The temperature of the heating block and CDL was set at 200 °C. The pressure of the drying gas was set to 115 kPa. The voltage of the detector and flow rate of the nebulizing gas were set as 1.75 kV and 1.5 L min^−1^, respectively.

The samples prepared in the “In vitro incubation of TYP and gut microbiota” section were added to three times the volume of methanol solution. After mixing, the samples were centrifuged at 4 °C and 13 000 rpm for 10 min, and 100 µL of the supernatant was collected for LC‐Q‐TOF determination.

### In Vitro Incubation of TYP Metabolites and the Gut Microbiota

For the metabolite group, 10 µL of each metabolite was added, and the final concentration of each metabolite was 10 µg mL^−1^ (Isorhamnetin‐3‐*O*‐nehesperidine, isorhamnetin‐3‐*O*‐nehesperidine, isorhamnetin, 3,4‐dihydroxyphenylacetic acid, protocatechuic acid, 3‐hydroxyphenylacetic acid). The same volume of methanol was administered to the control group, and the mixture was incubated for 24 h. The other conditions used were the same as those in Section “In vitro incubation of TYP and the gut microbiota.”

### Molecular Virtual Docking Analysis of TYP

Docking analyses of TYP, Rha, Glu, Flr, and Chi were conducted using Discovery Studio Client software. The crystal structures of Rha (PDB ID: 6i60), Glu (PDB ID: 8wfw), Flr (pdb: 7d39), and Chi (pdb: 3zph) were obtained from the Protein Data Bank (PDB) database. LibDock was selected for use in molecular virtual docking analysis of TYP.

### Pure Enzyme Metabolism Experiments

1 mg mL^−1^ Rha and Glu solutions was prepared by PBS solution.

For Rha metabolism experiments: 10 µL TYP solution (1 mg mL^−1^) was added into EP tubes and 990 µL Rha solutions was added. The mixture solutions were incubated at 37 °C. The incubation time was 0, 5, 10, 15, 30, 45, 60, 120 min. The sample was measured using the method described in section “Determination of the levels of TYP and TYP metabolites by LC‐MS/MS.”

For Glu metabolism experiments: 10 µL TYP solution (1 mg mL^−1^) was added into EP tubes and 990 µL Glu solutions was added. The mixture solutions were incubated at 37 °C. The incubation time was 0, 5, 10, 15, 30, 45, 60, 120 min. The sample was measured using the method described in section “Determination of the levels of TYP and TYP metabolites by LC‐MS/MS.”

### BglX Gene Deletions in *E. coli BL21(DE3)*

Recombinant arms and primers were designed at the 5′ and 3′ ends of the target gene. PCR amplified *kan* gene containing the homologous arm of bglX gene. Red recombinant enzyme plasmid pKD46 was transferred into *E. coli BL21(DE3)* to prepare electroreceptor state. Strain *E. coli BL21(DE3)* pKD46 was cultured at 30 °C, and when it grew to a suitable state, it was re‐suspended with 10% glycerol for three times.^[^ ^83^ ^]^ The appropriate amount of recombinant fragments was transferred according to the conditions recommended by the electroconverter, and after recovery, the plates were coated and cultured inversely at 30 °C. Positive clones were screened and purified by streaking, and plasmid pkd46 was eliminated. The knockout strain with cleared plasmids was inoculated to prepare electroreceptor state. Plasmid PCP20 was transferred into the knockout strain. After resuscitation, the plates were coated and cultured inversely at 30 °C. The clones were selected and identified as positive by PCR. After the positive streak was purified, the genome was sequenced by PCR. Plasmid PCP20 was cleared by correct bacteria. Plasmid PCP20 was eliminated by cultivating at 37 °C, and the knockout strain was transformed into a nonresistant knockout strain.

### Heterologous Expression of Bacteroides‐Derived Rhamnosidase in *E. coli* BL21(DE3)

The α‐l‐rhamnosidase gene (NZ_CP040530.1) from *B. thetaiotaomicron* was codon optimized for expression in *E. coli BL21(DE3)* ▵bglX. The rhamnosidase gene was amplified by PCR using primers, and the PCR product was digested with Nco I and XholI and inserted into pET‐28a at the Nco I and XholI sites to obtain the plasmid pET28a‐Rha. Plasmid pET28a‐Rha was transferred into *E. coli BL21(DE3)* ▵bglX. *E. coli BL21(DE3)* ▵bglX containing the α‐l‐rhamnosidase gene codon optimized sequence was cultured from glycerin solution at 37 °C and 220 rpm. Protein expression was induced by adding 0.5 × 10^−3^ m isopropyl β‐d‐1‐thiopyranogalactoside (IPTG) at various temperatures overnight until optical density (OD 600) at 600 nm reached 0.8–1.0.^[^ ^84^ ^]^ The recombinant enzyme was purified by Ni‐NTA affinity chromatography. Proteins were examined by polyacrylamide gel electrophoresis (SDS‐PAGE).

### In Vitro Incubation of TYP and Gut Microbiota from Pseudo‐Germ‐Free ob/ob Mice

Six ob/ob mice (male, 40–50 g) were orally treated with cefadroxil (100 mg kg^−1^), terramycin (300 mg kg^−1^), and erythromycin (300 mg kg^−1^) twice a day for 3 d. The intestinal contents of six mice were collected on the third day after the final treatment with antibiotics, and the PGF status was confirmed by culturing fecal samples in an anaerobic incubator on nutrient agar culture medium. The incubation process was the same as that in the “In vitro incubation of TYP and the gut microbiota” section.

### Colony Forming Units (CFU) Determination in Intestinal Contents of ob/ob Mice and Pseudo‐Germ‐Free ob/ob Mice

1.5% agar was added into LB medium and anaerobic medium, the medium was sterilized and poured onto plate. Intestinal contents in section “In vitro incubation of TYP and gut microbiota from pseudo‐germ‐free ob/ob mice” were weighed accurately and were mixed with sterile normal saline (w/v: 1/9). The mixture was mixed well and was diluted step by step according to 10^1^, 10^2^, 10^3^, and 10^4^. 10 µL of 10^4^ times dilution solution was taken and dripped onto the already prepared LB agar plate and anaerobic agar plate. The solution of control mice was dripped onto the left of the plate, the solution of PGF mice was dripped onto the right of the plate. Coating rod was used to evenly distribute the solution. LB agar plate was cultured at 37 °C for 24 h, under aerobic conditions. Anaerobic agar plate was cultured at 37 °C for 48 h, under anaerobic conditions.

### TYP was Metabolized by *F. plautii* In Vitro

*F. plautii* was cultured in Wilkens‐Chalgren Anaerobe (WCA) broth.^[^ ^37^ ^]^ After 72 h of cultivation, the bacterial strains were incubated with TYP (10 µg mL^−1^) under anaerobic conditions at 37 °C for 24 h. The concentration of TYP was determined at 0 min, 5 min, 10 min, 15 min, 30 min, 45 min, 1 h, 2 h, 4 h, 6 h, 12 h, and 24 h.

### TYP Was Metabolized by Four Individual Strains of Intestinal Bacteria In Vitro

*B. thetaiotaomicron* was cultured in brain heart infusion (BHI) medium. *B. uniformis* was cultured in ATCC 1490 medium. *A. muciniphila* was cultured in fluid thioglycollate medium. *L. rhamnosus GG* was cultured in MRS broth. After 48 h of cultivation, the bacterial strains were incubated with TYP (10 µg mL^−1^) under anaerobic conditions at 37 °C for 24 h. The concentration of TYP was determined at 0.5, 1, 2, 4, 6, 12, and 24 h.

### Determination of SCFA Levels

The quantitative method was modified based on a previously published method.^[^ ^49^ ^]^ GC was used for determination, and the chromatographic column used was a capillary column (AT‐WAX, 30 m × 0.25 mm × zero point two five µm. Alltech Company, ME) with a temperature gradient of 0–3 min, 80 °C, 3.01–13 min, 80–130 °C, 13.01–60 min, and 130 °C. The temperature of the detector and the injector was set to 250 and 230 °C, respectively.

For sample preparation, in the first experiment, the samples were divided into a control group and a TYP group, and the preparation process was similar to that described in the “In vitro incubation of TYP and gut microbiota” section. The TYP group was treated with a bacterial solution and TYP solution (concentration of TYP was 10 µg mL^−1^), while the control group was treated with a bacterial solution and methanol (concentration of methanol was 1%). The incubation time points were 6, 12, and 24 h. The second group was divided into a control group and a metabolite group. The preparation process was similar to that described earlier. The metabolites were isorhamnetin‐3‐*O*‐neohesperidin, isorhamnetin‐3‐*O*‐glucoside, isorhamnetin, 3,4‐dihydroxyphenylacetic acid, protocatechuic acid, and 3‐hydroxyphenylacetic acid. The final concentration of the metabolites was 10 µg mL^−1^. All the samples were mixed thoroughly with three volumes of acetonitrile (containing 1% phosphoric acid) and centrifuged for 10 min at 4 °C and 13 000 rpm. 100 µL of the supernatant was taken for GC determination.

### The Lipid Accumulation Model of HepG2 Cells

HepG2 cells were cultured in DMEM containing 100 U mL^−1^ penicillin, 100 U mL^−1^ streptomycin, and 10% fetal bovine serum at 37 °C and 5% CO_2_. HepG2 cells were inoculated into six‐well plates and divided into a control group, model group, TYP group, M1 group, M2 group, M3 group, M4 group, M5 group, M6 group, acetic acid group, propionic acid group, and butyric acid group. A lipid accumulation model of HepG2 cells was established by stimulating 0.5 × 10^−3^ m free fatty acids (with a palmitic acid:oleic acid ratio of 1:2) for 24 h.^[^ ^12^ ^]^ In the dosing groups, the concentration of the drugs was 50 × 10^−6^ m. TG, TC, and LDL‐C kits were subsequently used for determination.

### Effects of TYP, Bacterial Metabolites, and Butyrate on Hyperlipidemia In Vivo

Thirty ob/ob mice were divided into six groups: a model group (*n* = 5), a TYP group (*n* = 5), a TYP+PGF group (*n* = 5), M3 group (*n* = 5), M5 group (*n* = 5), and a butyrate group (*n* = 5). Five C57/6J mice were used as normal controls (*n* = 5). TYP group (100 mg kg^−1^ d^−1^), TYP+PGF group (TYP 100 mg kg^−1^ d^−1^, cefadroxil 100 mg kg^−1^ terramycin 300 mg kg^−1^, erythromycin 300 mg kg^−1^, the antibiotics were administrated twice a day), M3 group (100 mg kg^−1^ d^−1^), M5 group (100 mg kg^−1^ d^−1^), and butyrate group (100 mg kg^−1^ d^−1^). All drugs or natural products were administered orally for 21 d.

### Effects of Antibiotics on Hyperlipidemia In Vivo

Ten ob/ob mice were divided into two groups: a model group (*n* = 5) and a PGF group (*n* = 5). PGF group (cefadroxil 100 mg kg^−1^, terramycin 300 mg kg^−1^, erythromycin 300 mg kg^−1^, the antibiotics were orally administrated twice a day, for 21 d).

### Tight Junction Proteins Expression Analysis

Colon tissues of mice in each group were taken, weighed and put into a pre‐cooled centrifuge tube and cut into pieces on ice. The cut tissues were mixed with pre‐cooled PBS at 1:3 (weight g: volume mL), fully homogenized on ice with a homogenizer, centrifuged at 5000×*g* at 4 °C for 10 min, and the supernatant was taken for use. According to the kit instructions, the content of ZO‐1, Occludin, and Claudin‐5 in the colon of each group was quantitatively determined by commercial ELISA kits.

### LPS Analysis

The plasma of mice in each group was collected, and the LPS content in plasma of mice in each group was quantitatively determined by commercial ELISA kits according to the kit instructions.

### Fecal Microbiota Transplantation Experiment

For fecal transplantation experiment, five C57/6J mice were used as normal control (Group 1). 20 ob/ob mice (8 weeks, male) were randomly divided into four groups: Group 2 (model group); Group 3 (TYP group, 100 mg kg^−1^); Group 4, the fecal transplantation group treated with fecal bacteria from Group 3 (TYP→FMT group); Group 5, M3 and M5 were orally administered in combination (M3 100 mg kg^−1^, M5 100 mg kg^−1^). After adapting for 3 d, animals of Group 3 were orally treated with TYP for 21 d, animals of Group 3 were orally treated with M3 and M5 21 d. Three days after the end of administration, fresh feces from Group 3 were collected and stored at −80 °C for fecal microbiota transplantation. The fecal samples from Group 3 and were collected, thoroughly mixed with PBS (weight:volume = 1:9) and filtered to prepare the bacterial solution (once a day, 0.5 mL/100 g, for 21 d).

### Pharmacokinetics of TYP, M3 and M5 in C57BL/6 Mice

Three C57BL/6 mice were orally administered with TYP (100 mg kg^−1^). Three C57BL/6 mice were intravenously administered with TYP (5 mg kg^−1^). Three C57BL/6 mice were orally administered with M3 (100 mg kg^−1^). Three C57BL/6 mice were intravenously administered of M5 (5 mg kg^−1^). Three C57BL/6 mice were orally administered with M5 (100 mg kg^−1^). Three C57BL/6 mice were intravenously administered of M5 (5 mg kg^−1^). 60 µL blood from the orbital vein was collected into a heparinized tube at 0 min, 5 min, 10 min, 15 min, 30 min, 45 min, 1 h, 2 h, 4 h, 6 h, 10 h, and 24 h after drug administration. Plasma was collected after centrifugation at 5000 rpm and immediately stored at −80 °C before analysis.

### TYP and Metabolites Determination in Feces and Plasma

Feces of all groups in section “Effects of TYP, bacterial metabolites and butyrate on hyperlipidemia in vivo” were weighed accurately. Feces samples were added to three times the volume of PBS solution, the samples were crushed thoroughly, and mixed well. After mixing, the mixture was centrifuged at 4 °C and 13 000 rpm for 10 min, 100 µL supernatant was collected and mixed with 300 µL methanol solution containing internal standard. After mixing, the mixture was centrifuged at 4 °C and 13 000 rpm for 10 min, after which 100 µL of the supernatant was collected for LC‐MS/MS determination of the levels of TYP and its metabolites. Other procedures were the same with descriptions of section “Determination of the levels of TYP and TYP metabolites by LC‒MS/MS.”

30 µL plasma of TYP group in section “Effects of TYP, bacterial metabolites and butyrate on hyperlipidemia in vivo” were added into 90 µL methanol solution containing internal standard. After mixing, the mixture was centrifuged at 4 °C and 13 000 rpm for 10 min, after which 80 µL of the supernatant was collected for LC‐MS/MS determination of the levels of TYP and its metabolites. Other procedures were the same with descriptions of section “Determination of the levels of TYP and TYP metabolites by LC‐MS/MS.”

### 16S rRNA Gene Analysis

The V3‐4 hypervariable region of bacterial 16S rRNA gene was amplified with the universal primer 338F (5′‐ACTCCTACGGGAGGCAGCAG‐3′) and 806R (5′‐ GGACTACNNGGGTATCTAAT‐3′). The PCR products were purified using an Agencourt AMPure XP Kit (Beckman Coulter, Inc., USA). Sequencing libraries were generated using NEB Next Ultra II DNA Library Prep Kit (New England Biolabs, Inc., USA) following the manufacturer's recommendations. Deep sequencing was performed on Illumina Miseq/Novaseq (Illumina, Inc., USA) platform at Beijing Allwegene Technology Co., Ltd. After the run, image analysis, base calling, and error estimation were performed using Illumina Analysis Pipeline Version 2.6 (Illumina, Inc., USA). The raw data were divided into different samples according to the barcode sequence through QIIME (v1.8.0) software. Pear (v0.9.6) software was used to filter and splice raw data. The sequences were removed from consideration if they were shorter than 120 bp, had a low quality score (≤20), contained ambiguous bases. During splicing, the minimum overlap setting was 10 bp, and the mismatch rate was 0.1. After splicing, Vsearch (v2.7.1) software was used to remove sequences with length less than 230 bp and removed the chimeric sequence by uchime method according to the Gold Database. Qualified sequences were clustered into operational taxonomic units (OTUs) at a similarity threshold of 97% use Uparse algorithm of Vsearch (v2.7.1) software. To minimize the effect from sequencing depth to the intersample variation, samples were subsampled (rarefied) to XXXX sequences per sample by random sampling. The BLAST tool was used to classify all OTU representative sequences into different taxonomic groups against Silva138 Database, and e‐value threshold was set to 1 × 10^−5^. To describe the dissimilarity between multiple samples, PCA was analyzed by R (v3.6.0) based on the OTU information from each sample. The β‐Diversity distance matrix between samples was calculated using the Bray Curtis algorithms, and plotted PCoA or unweighted pair group method with arithmetic mean (UPGMA) clustering tree. Statistical analyses were conducted using two‐way ANOVA and Student's *t*‐test with GraphPad Prism Version 8 (GraphPad Software, La Jolla, CA).

### Metagenomic Analysis

DNA extract was fragmented to an average size of about 350 bp using Covaris M220 (Gene Company Limited, China) for paired‐end library construction. Paired‐end library was constructed using NEXTFLEX Rapid DNA‐Seq (Bioo Scientific, Austin, TX). Paired‐end sequencing was performed on Illumina NovaSeq X Plus (Illumina Inc., San Diego, CA) at Majorbio Bio‐Pharm Technology Co., Ltd. (Shanghai, China) using NovaSeq X Series 25B Reagent Kit according to the manufacturer's instructions (www.illumina.com). Briefly, the raw sequencing reads were trimmed of adapters, and low‐quality reads (length < 50 bp or with average quality value <20) were removed by fastp (https://github.com/OpenGene/fastp, version 0.20.0). The quality‐filtered data were assembled using MEGAHIT[3] (https://github.com/voutcn/megahit, version 1.1.2). Contigs with a length ≥300 bp were selected as the final assembling result. Open reading frames (ORFs) from each assembled contigs were predicted using Prodigal (https://github.com/hyattpd/Prodigal, version 2.6.3) and a length ≥100 bp ORFs were retrieved.

A nonredundant gene catalog was constructed using CD‐HIT (http://weizhongli‐lab.org/cd‐hit/, version 4.7) with 90% sequence identity and 90% coverage. Gene abundance for a certain sample was estimated by SOAPaligner (https://github.com/ShujiaHuang/SOAPaligner, version soap2.21 release) with 95% identity.

The best‐hit taxonomy of nonredundant genes was obtained by aligning them against the NCBI NR database by DIAMOND (http://ab.inf.uni‐tuebingen.de/software/diamond/, version 2.0.13). Similarly, the functional annotation (GO, KEGG, eggNOG, CAZy, CARD, PHI) of nonredundant genes was obtained. Based on the taxonomic and functional annotation and the abundance profile of nonredundant genes, statistical analyses were conducted using one‐way ANOVA and Student's *t*‐test with GraphPad Prism Version 8 (GraphPad Software, La Jolla, CA)

### Statistical Analysis

Statistical analyses were conducted using two‐way ANOVA and Student's *t*‐test with GraphPad Prism Version 8 (GraphPad Software, La Jolla, CA). The data were expressed as the means ± standard deviation. *p*‐values less than 0.05 were considered statistically significant. DAS 2.0 was used to calculate the plasma pharmacokinetic parameters.

## Conflict of Interest

The authors declare no conflict of interest.

## Supporting information



Supporting Information

## Data Availability

The data that support the findings of this study are available from the corresponding author upon reasonable request.
