# Supplementary material for: Multiple Enzymes Expressed by the Gut Microbiota Can Transform Typhaneoside and Are Associated with Improving Hyperlipidemia
Source: Adv Sci (Weinh). 2025 Jan 22;12(10):2411770. doi: 10.1002/advs.202411770 (PMC11904961; doi:10.1002/advs.202411770)
Supplement: Supplementary file 1 — Supporting Information [file ADVS-12-2411770-s001.docx]

Supporting Information

Multiple enzymes expressed by the gut microbiota can transform typhaneoside and are associated with improving hyperlipidemia

Hui Xu^1,#^, Ru Feng^1,#^, Meng-Liang Ye^1,#^, Jia-Chun Hu^1^, Jin-Yue Lu^1^, Jing-Yue Wang^1^, Heng-Tong Zuo^1^, Yi Zhao^1^, Jian-Ye Song^1^, Jian-Dong Jiang^1, *^, Yun-Zhi Zhou^2, *^, Yan Wang^1, *^

1. State Key Laboratory of Bioactive Substance and Function of Natural Medicines, Institute of Materia Medica, Chinese Academy of Medical Sciences/Peking Union Medical College, Beijing 100050, China

2. Emergency General Hospital, National Research Center for Emergency Medicine, Beijing 100028, China

*Corresponding authors:

Dr Jian-Dong Jiang, e-mail address: jiang.jdong@163.com

Dr Yun-Zhi Zhou, e-mail address: [zhouyunzhi2017@126.com](mailto:zhouyunzhi2017@126.com)

Dr Yan Wang, e-mail address: [wangyan@imm.ac.cn](mailto:wangyan@imm.ac.cn)

^#^Equal contributions to the manuscript

This PDF file includes:

Figures S1-S9

Figure legends of Figures S1-S9


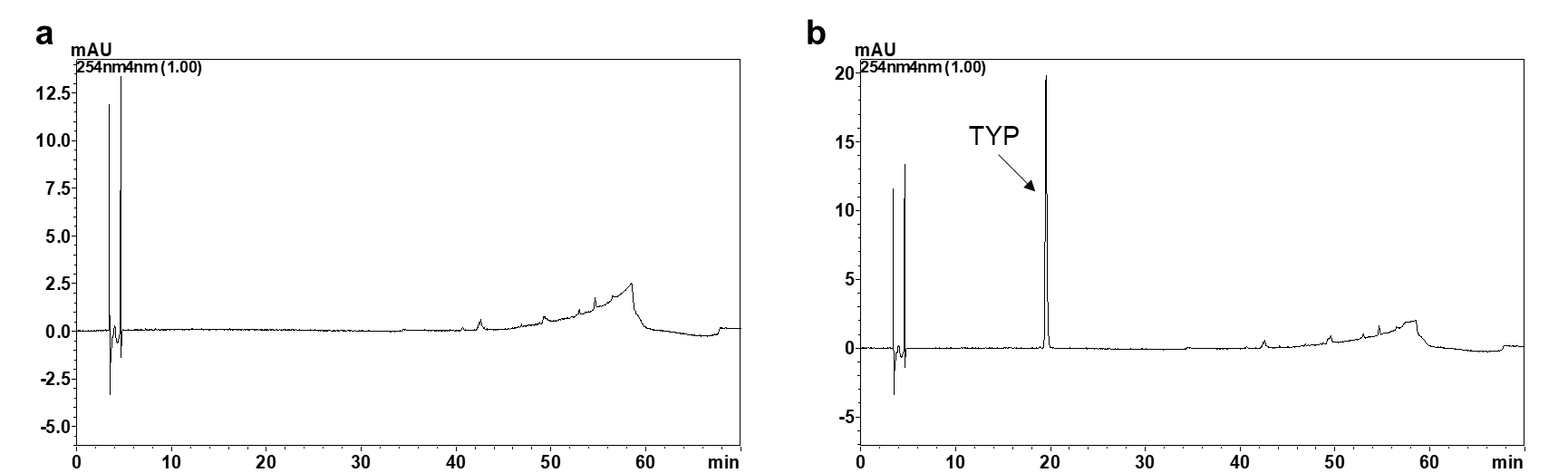


**Figure S1**. a) Chromatogram of blank solvent methanol. b) Chromatogram of TYP standard


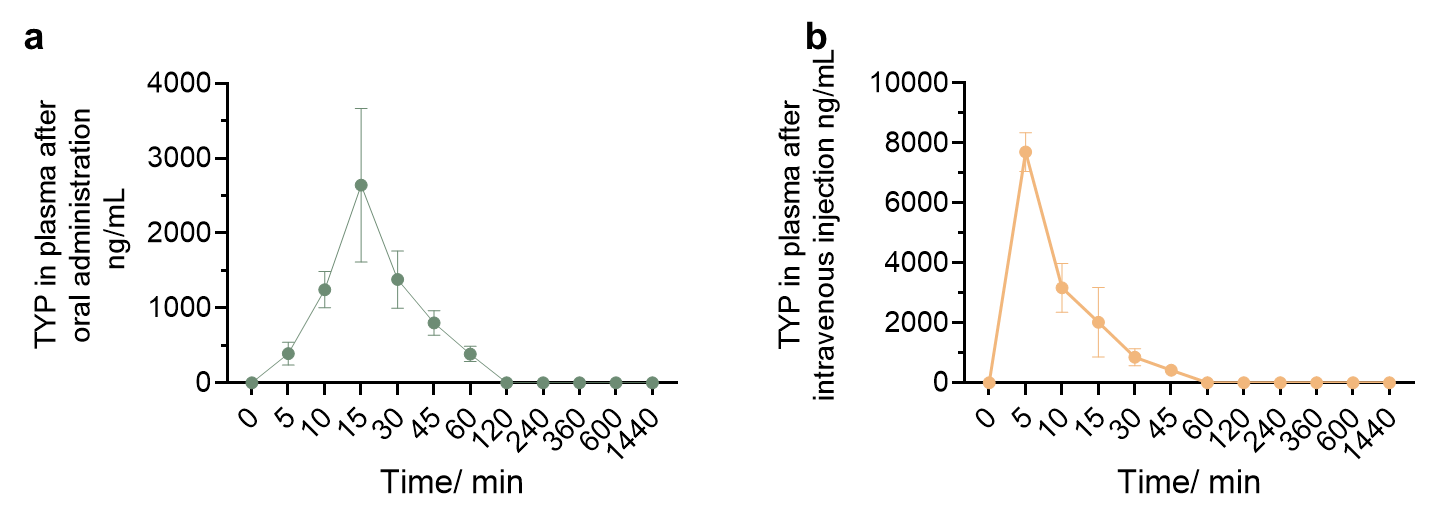


**Figure S2**. Pharmacokinetic curve of TYP, M3 and M5. a) Mean plasma concentration-time profiles of TYP after oral administration. b) Mean plasma concentration-time profiles of TYP after intravenous injection.

*
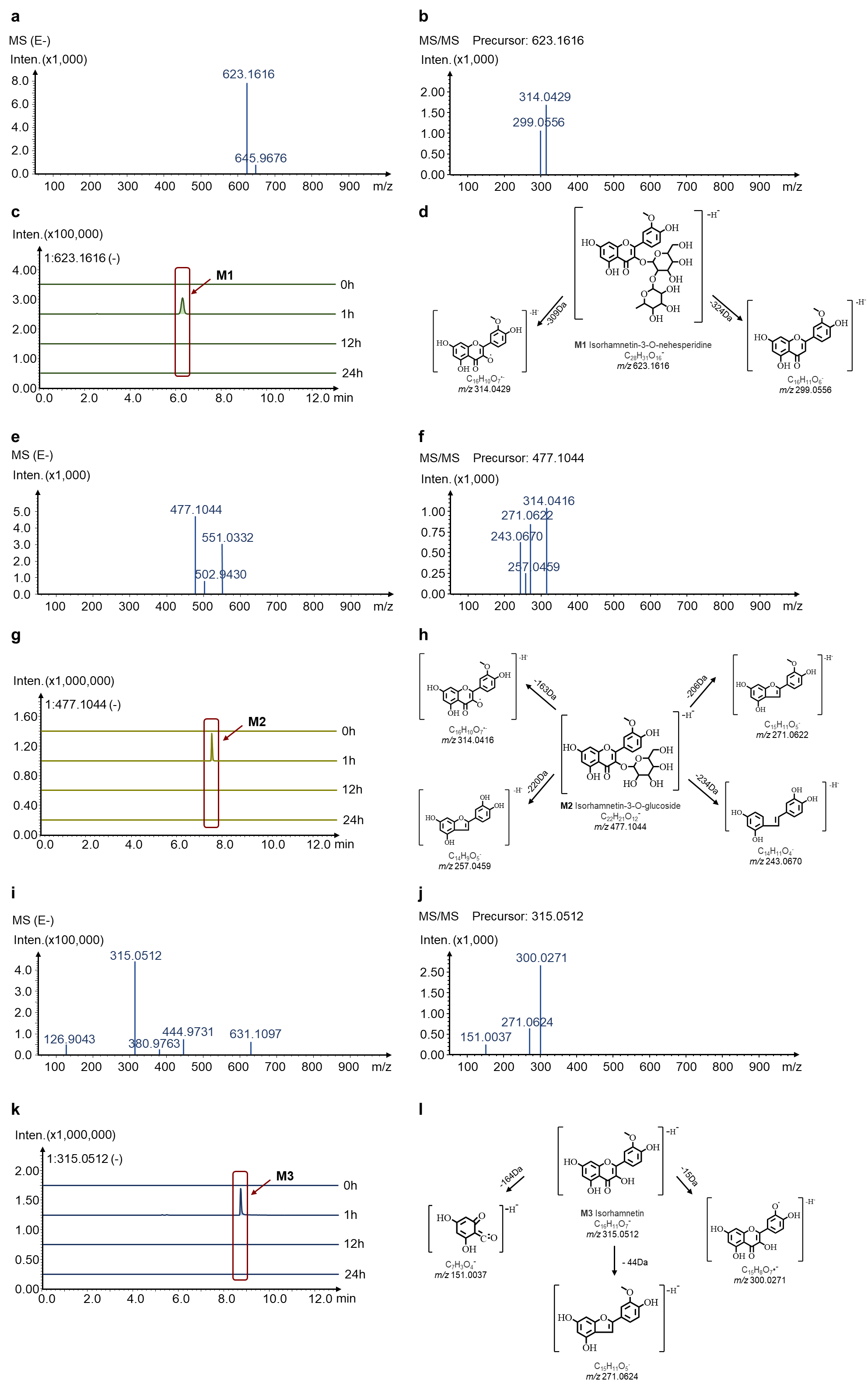
*

**Figure S3**. TYP was metabolized into three intermediate microbiota metabolites. a) MS spectrogram of M1. b) MS/MS spectrogram of M1. c) EIC of M1 after different incubation times. d) Hypothesized mass spectrometric cleavage pathway of M1. e) MS spectrogram of M2. f) MS/MS spectrogram of M2. g) EIC of M2 after different incubation times. h) Hypothesized mass spectrometric cleavage pathway of M2. i) MS spectrogram of M3. j) MS/MS spectrogram of M3. k) EIC of M3 after different incubation times. l) Hypothesized mass spectrometric cleavage pathway of M3.


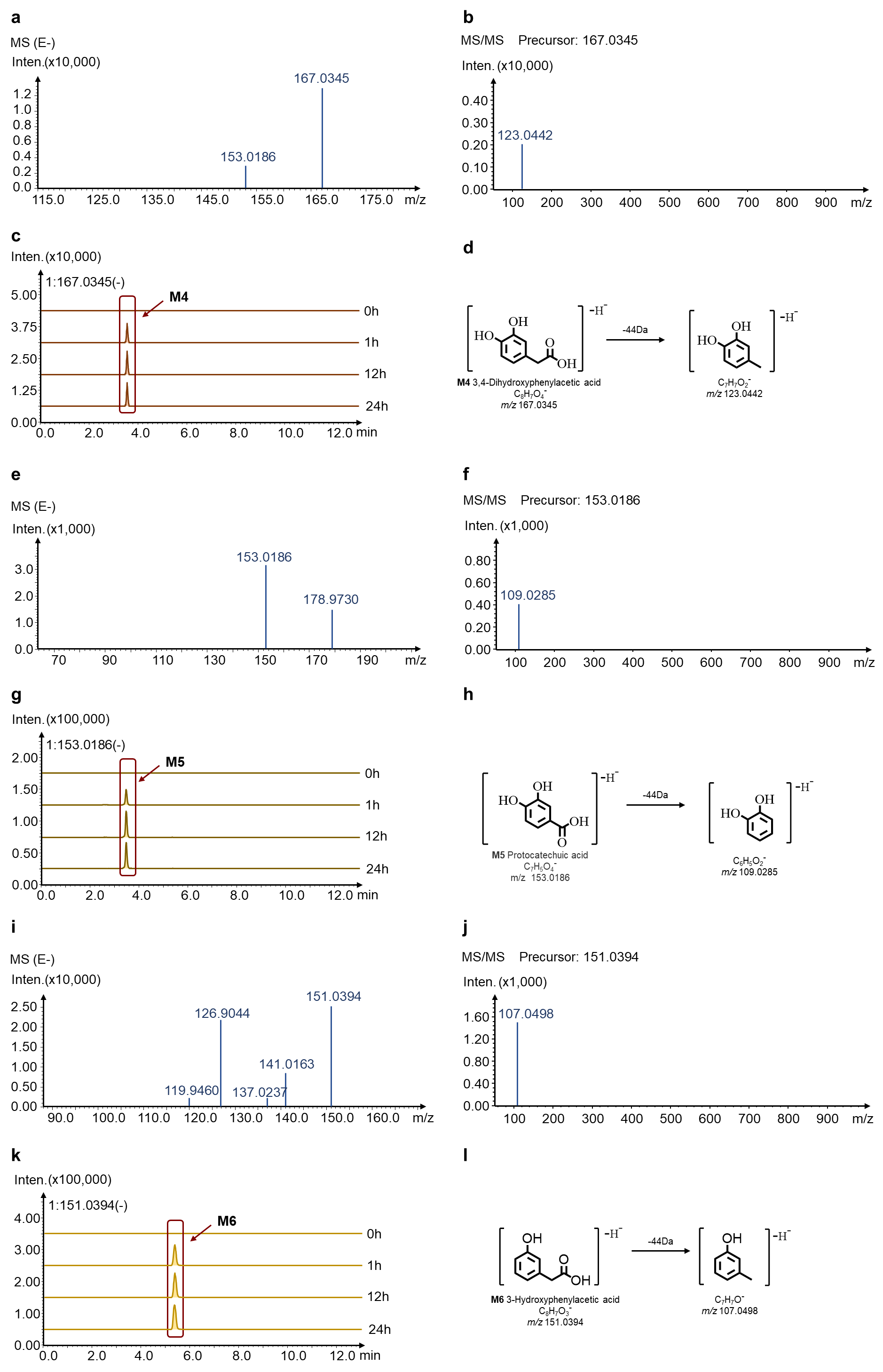


**Figure S4**. TYP was metabolized into three final microbiota-derived metabolites. a) MS spectrogram of M4. b) MS/MS spectrogram of M4. c) EIC of M4 after different incubation times. d) Hypothesized mass spectrometric cleavage pathway of M4. e) MS spectrogram of M5. f) MS/MS spectrogram of M5. g) EIC of M5 after different incubation times. h) Hypothesized mass spectrometric cleavage pathway of M5. i) MS spectrogram of M6. j) MS/MS spectrogram of M6. k) EIC of M6 after different incubation times. l) Hypothesized mass spectrometric cleavage pathway of M6.


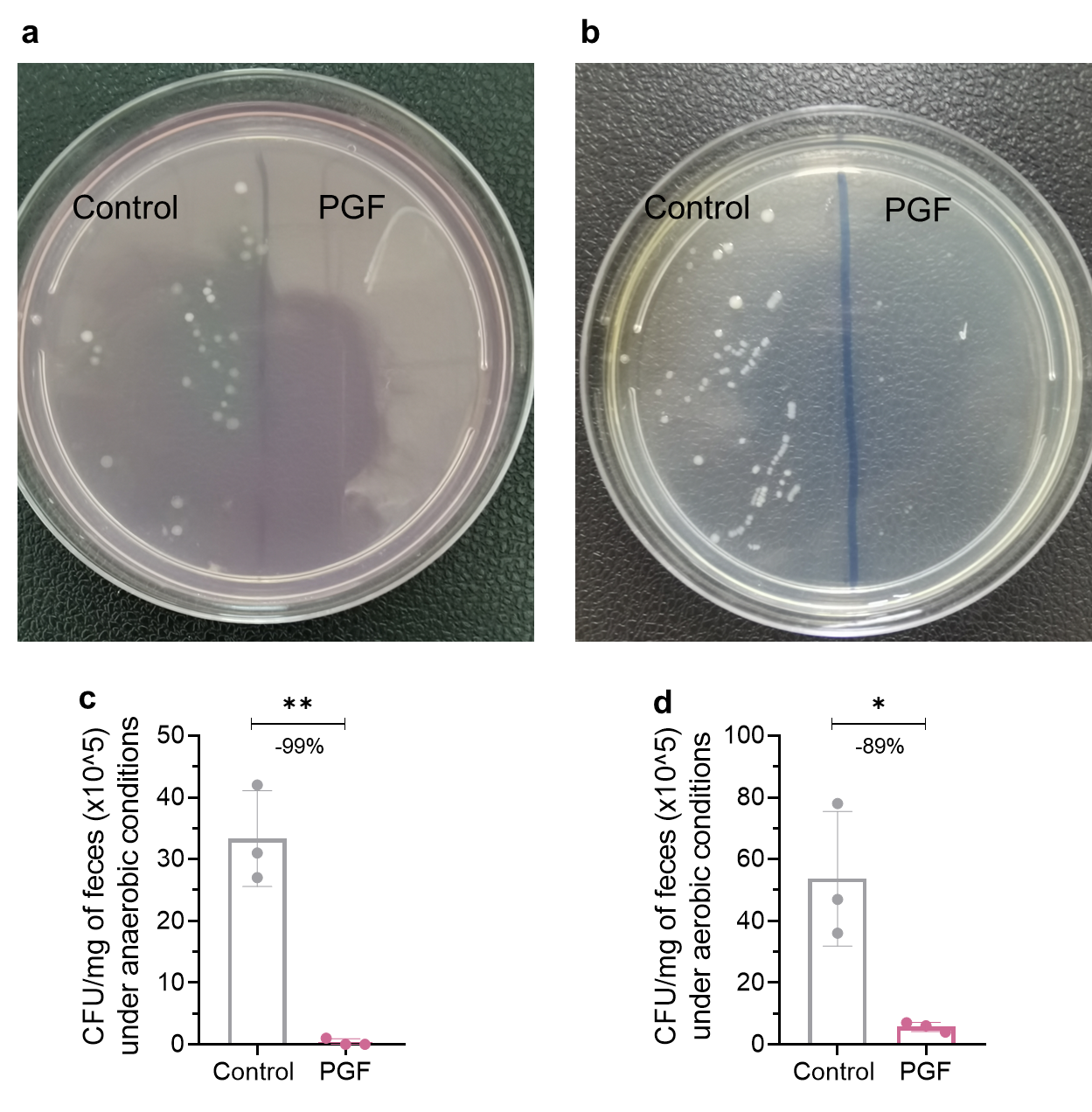


Figure S5 Pseudo germ-free (PGF) ob/ob mice were established through oral antibiotics a) Representative figure of a plate colony under anaerobic conditions. b) Representative picture of a plate colony under aerobic conditions. c) CFU/mg of feces (x10^5) under anaerobic conditions. d) CFU/mg of feces (x10^5) under aerobic conditions.


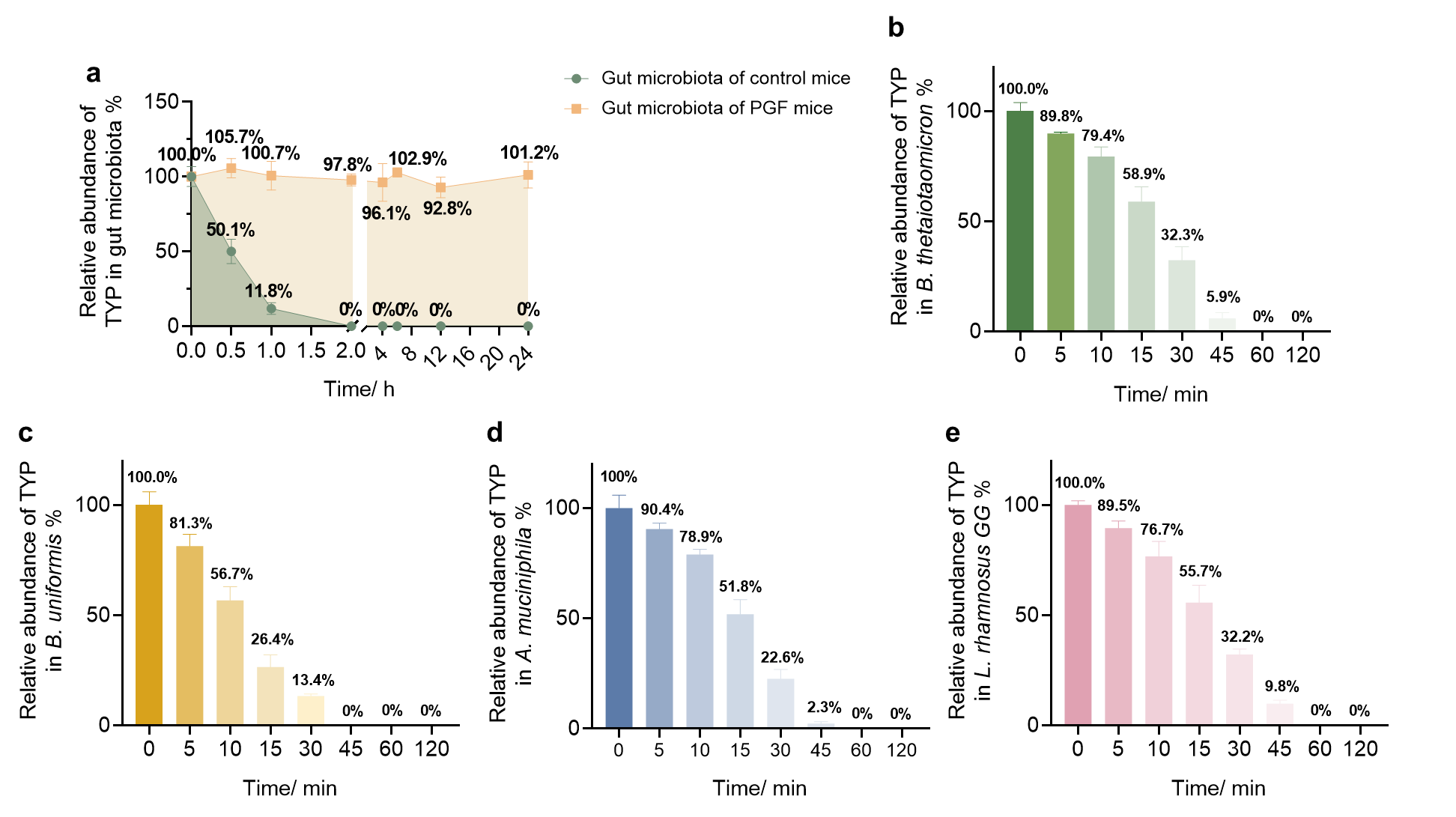


Figure S6. TYP was metabolized by four individual strains of intestinal bacteria *in vitro.* a) Relative abundance of TYP in the gut microbiota of ob/ob mice and PGF ob/ob mice. b) Relative abundance of TYP after incubating with B. thetaiotaomicron. c) Relative abundance of TYP after incubating with *B. uniformis.* d) Relative abundance of TYP after incubating with *A. muciniphila*. e) Relative abundance of TYP after incubating with *L. rhamnosus.*


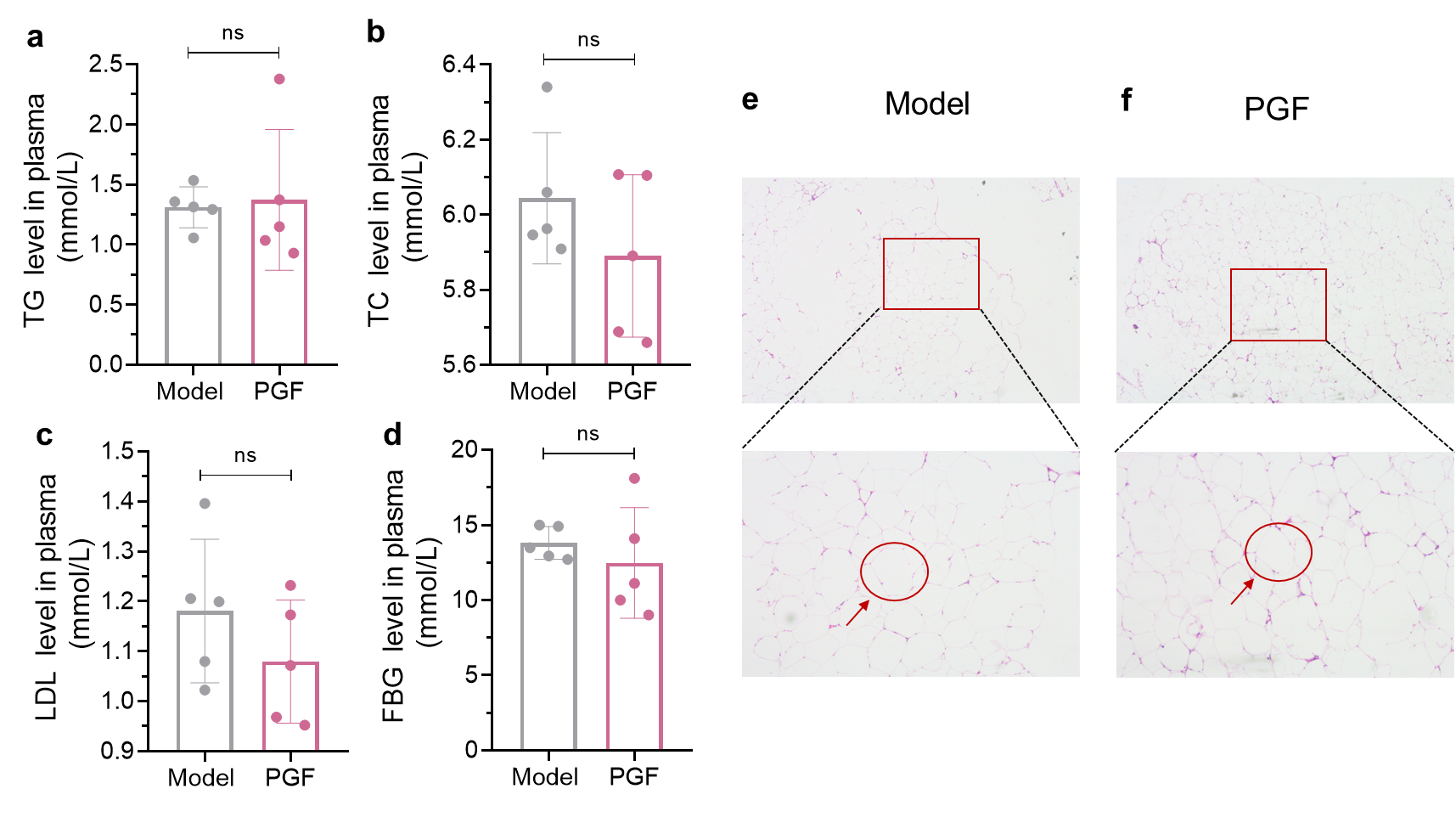


Figure S7 Antibiotics showed no effect on Model group. a) TG levels in plasma. b) TC levels in plasma. c) LDL-C levels in plasma. d) Fasting blood glucose (FBG) levels in plasma. e-f) The staining of epididymal white adipose tissue (eWAT) with H&E . The cell framed in red circles and pointed by red arrows is white fat cell. ns: no significance.


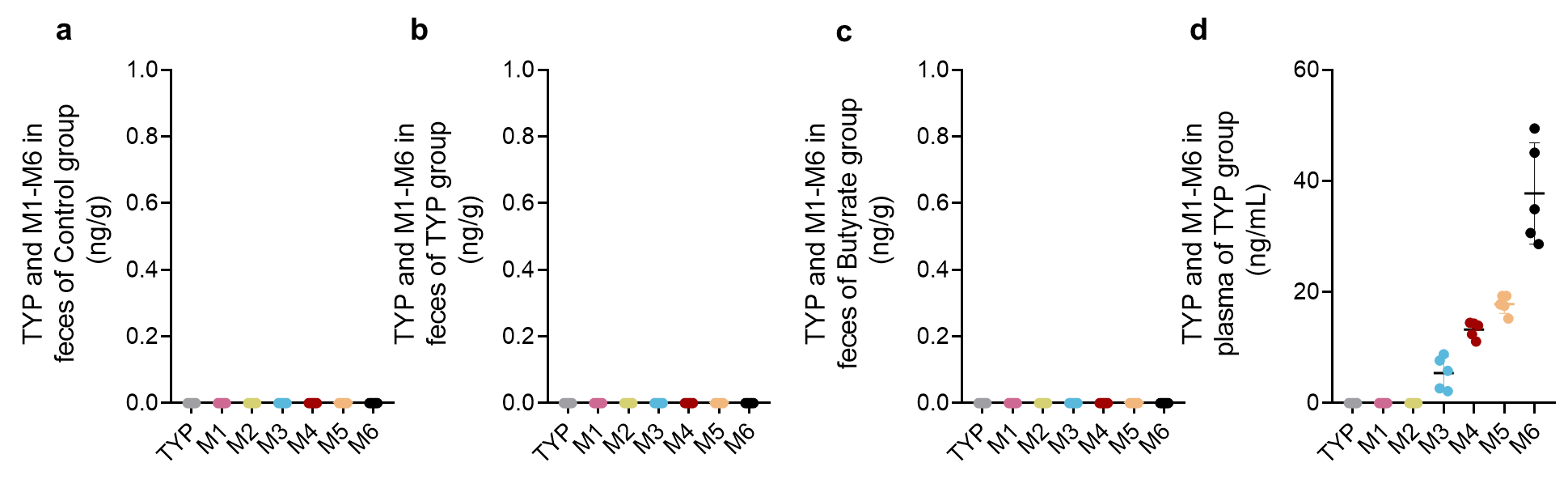


Figure S8 TYP and M1-M6 levels in feces and plasma. a) TYP, M1-M6 level in feces of control group. b) TYP, M1-M6 level in feces of Model group. c) TYP, M1-M6 level in feces of Butyrate group. d) TYP, M1-M6 level in plasma of TYP group.


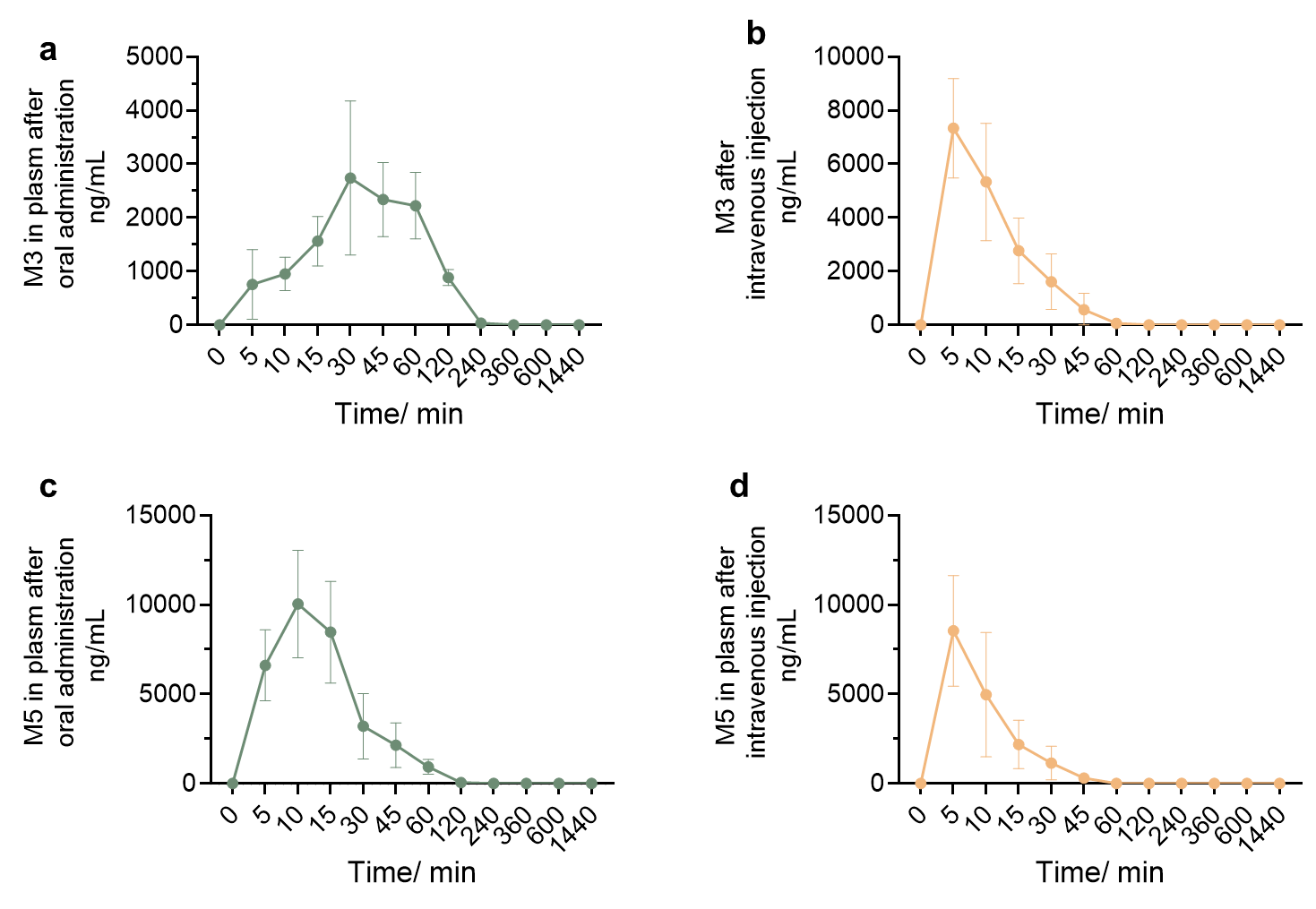


Figure S9. Pharmacokinetic curve of M3 and M5. a) Mean plasma concentration-time profiles of M3 after oral administration. b) Mean plasma concentration-time profiles of M3 after intravenous injection. c) Mean plasma concentration-time profiles of M5 after oral administration. d) Mean plasma concentration-time profiles of M5 after intravenous injection.
